# Phospholipases of Mineralization Competent Cells and Matrix Vesicles: Roles in Physiological and Pathological Mineralizations

**DOI:** 10.3390/ijms14035036

**Published:** 2013-03-01

**Authors:** Saida Mebarek, Abdelkarim Abousalham, David Magne, Le Duy Do, Joanna Bandorowicz-Pikula, Slawomir Pikula, René Buchet

**Affiliations:** 1 Université de Lyon, Lyon, F-69361, France; E-Mails: abousalham@univ-lyon1.fr (A.A.); david.magne@univ-lyon1.fr (D.M.); ldo@nencki.gov.pl (L.D.D.); Rene.Buchet@univ-lyon1.fr (R.B.); 2 Université Lyon 1, Villeurbanne, F-69622, France; 3 INSA-Lyon, Villeurbanne, F-69622, France; 4 CPE Lyon, Villeurbanne, F-69616, France; 5 ICBMS CNRS UMR 5246, Villeurbanne, F-69622, France; 6 Department of Biochemistry, Nencki Institute of Experimental Biology, Polish Academy of Sciences, Pasteura 3, 02-093 Warsaw, Poland; E-Mails: j.bandorowicz-pikula@nencki.gov.pl (J.B.-P.); slawek@nencki.gov.pl (S.P.)

**Keywords:** bone, cartilage, osteoarthritis, osteoporosis, phospholipases, rheumatoid arthritis, sphingomyelinase, osteoblasts, osteoclasts, chondrocytes, Smooth muscle cells, matrix vesicle, mineralization, vascular calcification

## Abstract

The present review aims to systematically and critically analyze the current knowledge on phospholipases and their role in physiological and pathological mineralization undertaken by mineralization competent cells. Cellular lipid metabolism plays an important role in biological mineralization. The physiological mechanisms of mineralization are likely to take place in tissues other than in bones and teeth under specific pathological conditions. For instance, vascular calcification in arteries of patients with renal failure, diabetes mellitus or atherosclerosis recapitulates the mechanisms of bone formation. Osteoporosis—a bone resorbing disease—and rheumatoid arthritis originating from the inflammation in the synovium are also affected by cellular lipid metabolism. The focus is on the lipid metabolism due to the effects of dietary lipids on bone health. These and other phenomena indicate that phospholipases may participate in bone remodelling as evidenced by their expression in smooth muscle cells, in bone forming osteoblasts, chondrocytes and in bone resorbing osteoclasts. Among various enzymes involved, phospholipases A_1_ or A_2_, phospholipase C, phospholipase D, autotaxin and sphingomyelinase are engaged in membrane lipid remodelling during early stages of mineralization and cell maturation in mineralization-competent cells. Numerous experimental evidences suggested that phospholipases exert their action at various stages of mineralization by affecting intracellular signaling and cell differentiation. The lipid metabolites—such as arachidonic acid, lysophospholipids, and sphingosine-1-phosphate are involved in cell signaling and inflammation reactions. Phospholipases are also important members of the cellular machinery engaged in matrix vesicle (MV) biogenesis and exocytosis. They may favour mineral formation inside MVs, may catalyse MV membrane breakdown necessary for the release of mineral deposits into extracellular matrix (ECM), or participate in hydrolysis of ECM. The biological functions of phospholipases are discussed from the perspective of animal and cellular knockout models, as well as disease implications, development of potent inhibitors and therapeutic interventions.

## Contents

1. Introduction 5040
  1.1. Bone Biology and Physiological Mineralization 5040
  1.2. Ectopic Calcifications and Defective Mineralizations 5042
  1.3. Matrix Vesicles and Early Stages of Mineralization 5043
  1.4. Dietary Lipids and Bone Health 5044
  1.5. Groups of Phospholipases and Possible Roles during Mineralization 5044
2. Phospholipases A_1_ 5047
  2.1. Groups, Subgroups and Specificity 5047
3. Phospholipases A_2_ 5048
  3.1. Groups, Subgroups and Specificity 5048
  3.2. Presence of PLA_2_s in Chondrocytes and Possible Roles 5049
  3.3 Presence of PLA_2_s in Osteoblasts and Possible Roles 5050
  3.4. Presence of PLA_2_s in osteoclasts and Possible Roles 5051
  3.5. Presence of PLA_2_s in Smooth Muscle Cells and Possible Roles 5052
  3.6. The Expressions of PLA_2_s under Pathological Conditions 5052
  3.7. Transgenic Knockout Animal for PLA_2_ Enzymes as Models for Bone Formation and Mineralization Diseases 5053
  3.8. Inhibitors of PLA_2_ as Drug Therapy 5053
  3.9. Effects Mediated by Arachidonic Acid and Its Pathways at Cellular Level 5054
    3.9.1. Effects Mediated by PGE_2_ 5055
    3.9.2. Effects Mediated by PGF_2_α and PGD_2_ 5056
  3.10. Effects Mediated by Lysophospholipids and Their Pathways at Cellular Level 5057
  3.11. The Effects of PLA Metabolites at Matrix Vesicle Level 5060
4. Non-Specific Phospholipase C 5060
  4.1. Groups, Subgroups and Specificity 5060
  4.2. Presence of PC-PLC in Chondrocytes and in Osteoblasts and Its Possible Role 5061
  4.3. Presence of PC-PLC in Osteoclasts and Possible Roles 5061
  4.4. Presence of PC-PLC in Smooth Muscle Cells and Possible Roles 5061
  4.5. The Effect of PLC Metabolites in Matrix Vesicles 5061
5. PI-Specific Phospholipase C 5062
  5.1. Groups, Subgroups and Specificity 5062
  5.2. PI-PLC in Tissues 5063
  5.3. Presence of PI-PLC in Chondrocytes and Possible Roles. 5064
  5.4. Presence of PI-PLC in Osteoblasts 5065
    5.4.1. Endothelin-1 Induced Signaling Pathway 5065
    5.4.2. Basic FGF Induced Signaling Pathway 5066
    5.4.3. Platelet-Derived Growth Factor Induced Signaling Pathway 5066
    5.4.4. Parathyroid Hormone Induced Signaling Pathway 5066
    5.4.5. PGD_2_ Induced-Signaling Pathway 5066
    5.4.6. PGE_2_ Induced-Signaling Pathway 5067
    5.4.7. PGF_2_ Induced-Signaling Pathway 5067
    5.4.8. Vitamin D-Induced Signaling Pathway 5067
    5.4.9. Interleukin-1-Induced Signaling Pathway 5067
    5.4.10. Miscelanous Ligand Binding Stimulated PI-PLC in Osteoblasts 5068
    5.4.11. Purinergic and Serotonin-2 B Receptors 5068
  5.5. Presence of PI-PLC in Osteoclasts 5068
    5.5.1. Calcitonin Induced Signaling Pathway 5069
    5.5.2. Intracellular Ca^2+^ Induced Signaling Pathway 5069
    5.5.3. Osteoprotegrin Induced Signaling Pathway 5070
    5.5.4. RANK Induced Signaling Pathways 5071
    5.5.5. Parathyroid Hormone Induced Signaling Pathway 5071
  5.6. Presence of PI-PLC in Smooth Muscle Cells and Possible Roles 5072
  5.7. Presence of PI-PLC in Odontoblasts and Possible Roles 5072
  5.8. Genetic Models 5073
6. PLC-Related but Catalytically Inactive Protein 5073
7. Sphingomyelinase 5074
  7.1. Groups, Subgroups and Specificity 5074
  7.2. Presence of Sphingomyelinase in Chondrocytes and Possible Roles 5074
  7.3. Presence of Sphingomyelinase in Osteoblasts and Possible Roles 5074
  7.4. Presence of Sphyngomyelinase in Osteoclasts and Possible Roles 5075
  7.5. Genetic Models 5075
  7.6. Effects of Sphyngomyelinase Metabolites at Matrix Vesicle Level 5075
8. Phospholipase D 5076
  8.1. Groups, Subgroups and Specificity 5076
  8.2. Presence of PLD in Chondrocytes and Possible Roles 5077
  8.3. Presence of PLD in Osteoblasts and Possible Roles 5079
  8.4. Presence of PLD in Osteoclasts and Possible Roles 5080
  8.5. Genetic Models 5080
  8.6. Effects of PLD Metabolite at Matrix Vesicle Level 5080
9. Non-HKD Enzymes—GPI-PLD 5081
  9.1. Groups, Subgroups and Specificity 5081
  9.2. Presence of GPI-PLD in Chondrocytes and Possible Roles 5081
  9.3. Presence of GPI-PLD in Osteoblasts and Possible Roles 5082
10. Non-HKD Enzymes—Autotaxin 5082
  10.1. Groups, Subgroups and Specificity 5082
  10.2. Presence of ATX in Chondrocytes and Possible Roles. 5083
  10.3. Presence of ATX in Osteoblasts and Possible Roles 5083
  10.4. Presence of ATX in Osteoclasts and Possible Roles. 5084
  10.5. Presence of ATX in Smooth Muscle Cells and Possible Roles 5084
11. Concluding Remarks 5084
Acknowledgments 5084
References 5084

## 

Abbreviations

1α,25-(OH)_2_D_3_: 1α,25-dihydroxyvitamin D_3_
24R,25(OH)_2_D_3_: 24R,25-dihydroxyvitamin D_3_
AA: arachidonic acid
ATX: autotaxin
BM: bone marrow
Ca^2+^_e_: extracellular Ca^2+^
Ca^2+^_i_: intracellular Ca^2+^
CaR: calcium-sensing receptor
CIA: collagen-induced arthritis
cPLA_2_: cytosolic Ca^2+^-dependent PLA_2_
COX: cyclooxygenase
DAG: diacylglycerol
DHT: 5α-dihydrotestosterone
ECM: extracellular matrix
ERK: extracellular signal-regulated kinase
ET: endothelin
FGF: fibroblast growth factor
GPCR: G-protein-coupled receptor
GPI-PLD: glycosyl-PI specific PLD
HA: hydroxyapatite
IL: interleukin
IP_3_: inositol 1,4,5-trisphosphate
iPLA_2_: Ca^2+^-independent PLA_2_
LOX: lipooxygenase
LPA: lysophosphatidic acid
LPC: lysophosphatidylcholine
LPE: lysophosphatidylethanolamine
LPG: lysophosphatidylglycerol
LPI: lysophosphatidylinositol
LPL: lysophospholipid
LPS: lysophosphatidylserine
LRRc17: leucine-rich repeat-containing 17
MAP: mitogen activated protein
MPP: metalloproteinase
MV: matrix vesicle
NF-κB: nuclear factor κB
NFAT: nuclear factor of activated T cell
NPP: ectonucleotide pyrophosphatase phosphodiesterase
NSAID: non-steroidal anti-inflammatory drug
OA: osteoarthritis
OPG: osteoprotegerin
PA: phosphatidic acid
PAF: platelet-activating factor
PAF-AH: PAF-acetylhydrolase
PBMC: peripheral blood mononuclear cell
PC: phosphatidylcholine
PChol: phosphocholine
PE: phosphatidylethanolamine
PEA: phosphoethanolamine
PGD_2_: prostaglandin D2
PGE_1_: prostaglandin E1
PGE_2_: prostaglandin E2
PGF_2_: prostaglandin F2
PH: pleckstrin homology
PHOSPHO1: phosphatase orphan 1
PI: phosphatidylinositol
PI-PLC: PI-specific
PIP_2_: PI 4,5-bisphosphate
PIP_3_: PI 3,4,5-trisphosphate
PKC: protein kinase C
PLA_1_: phospholipase A_1_
PLA_2_: phospholipase A_2_
PLC: phospholipase C
PLD: phospholipase D
P_i_: inorganic phosphate
PP_i_: inorganic pyrophosphate
PRIP: PLC-related but catalytically inactive protein
PS: phosphatidylserine
PS-PLA_1_: PS-specific PLA_1_
PTH: parathyroid hormone
PTX: pertussis toxin
PUFA: polyunsaturated fatty acid
RA: rheumatoid arthritis
RANKL: receptor activator of nuclear factor κB ligand
Runx2: runt-related transcription factor 2
SH: src homology
SM: sphingomyelin
SMase: sphingomyelinase
SMPD3: sphingomyeline phosphodiesterase-3
sPLA_2_: secreted PLA_2_
S1P: sphingosine-1-phosphate
STAT: signal transducer and activator of transcription
TNAP: tissue-non specific alkaline phosphatase
TNF: tumor necrosis factor
VSMC: vascular smooth muscle cell

## 1. Introduction

### 1.1. Bone Biology and Physiological Mineralization

**Figure 1:**
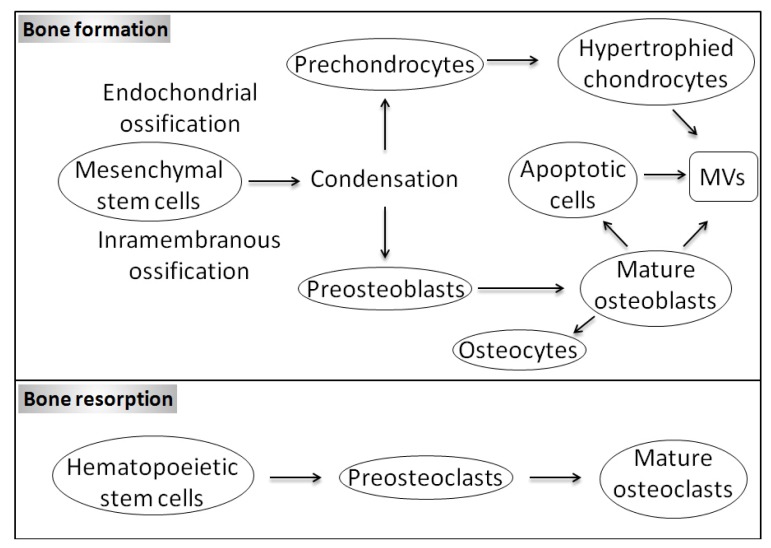
Schematic representation of bone formation and resorption. Bone formation: During endochondral ossification: chondrocytes first synthesize a cartilage. Central chondrocytes undergo hypertrophic differentiation and then undergo apoptose inducing vascular invasion and formation of a primary ossification centre. During intramembranous ossification, mesenchyme condensations differentiate into osteoblasts, which synthesize and mineralize osteoid to form a new bone without the requirement for a cartilage intermediate. A large fraction of mature osteoblasts undergo to apoptosis while a small fraction of mature osteoblasts become osteocytes. Bone resorption: Hematopoietic stem cells of the monocyte/macrophage lineage differentiate to mature osteoclasts and resorb bone.

The extracellular matrix (ECM) mineralization is a physiological process occurring in bone and teeth during skeletal growth in growth plate cartilage. In these tissues, this process is maintained by mineralization-competent cells, e.g., osteoblasts, odontoblasts, and hypertrophic chondrocytes. Bone formation begins when mesenchymal cells form condensations (Figure 1). During intramembranous ossification, which occurs in a few areas such as the flat bones of the skull, cells present in these condensations differentiate directly into bone-forming osteoblasts producing a matrix particulary rich in collagen I. At the end of the bone formation phase, osteoblasts may be subjected to apoptosis, become inactive osteoblasts, bone lining cells or osteocytes (Figure 1) [1]. The proportion of osteoblasts following each fate is not the same in all mammals and is not conserved among all types of bone [2]. In human cancellous bone, 65% of the osteoblasts undergo apoptosis and only about 30% transform into osteocytes [3], while in the antlers of the white-tailed deer (*Odocoileus virginianus*) 10% osteoblasts transform into osteocytes [4]. In advanced bony fishes with acellular bone, the number of osteoblasts that turn into osteocytes is zero [5,6]. During endochondral ossification, which occurs in most condensations, the cells become chondrocytes (Figure 1), *i.e.*, the primary cell type of cartilage, which secretes a matrix rich in collagen II [7]. Subsequently, chondrocytes in the centre of cartilage mould stop proliferating, become hypertrophic and start to produce collagen X. The hypertrophic chondrocytes are the principal engine of bone growth [8]. Hypertrophic chondrocytes direct the mineralization of the surrounding matrix, attract blood vessels and attract chondroclasts (closely related or identical to osteoclasts) [7]. These cells direct adjacent perichondrial cells to become osteoblasts. Finally, hypertrophic chondrocytes undergo apoptotic cell death [7]. Osteoblasts, lining cells and osteoclasts on the surface of bone as well as osteocytes in the interior of the bone are the four different types of bone cells (Figure 1). Osteoblasts originate from local mesenchymal cells called osteoprogenitor cells, while osteoclasts originate from bone marrow (BM) hematopoietic stem cells. Bone is a dynamic tissue that is constantly being reshaped by osteoblasts, which are in charge of matrix and mineral production, and by osteoclasts, which have long been recognized as cells that resorb the bone in normal bone remodelling and in pathological conditions when bone resorption is increased [9]. In fact, the mechanisms of mineralization used by mineralization-competent cells are very similar to each other. First, mineralization-competent cells differentiate under the control of the runt-related transcription factor 2 (Runx2). Then, they are able to secrete ECM, principally composed of fibrillar collagen, in which the calcium phosphate crystals with the hydroxyapatite (HA) structure are deposited [10]. The initiation of formation of calcium phosphate deposits is likely to start at matrix vesicles (MVs) [11], which are then released by the mineralization-competent cells into ECM. MVs are spherical bodies in 50–200 nm in diameter [11], enriched in tissue-non specific alkaline phosphatase (TNAP), which is indispensable for mineralization [12]. It was established a long time ago that inorganic pyrophosphate (PP_i_) or polyphosphate must be removed from the sites of mineralization, before calcification can occur [13]. On the other hand, it was commonly thought until recently that main function of TNAP is to generate inorganic phosphate (P_i_) to sustain mineral formation. This discrepancy has been solved by demonstrating that TNAP initiates mineralization by hydrolysing PP_i_ to P_i_, therefore removing PP_i_ which is a strong mineralization inhibitor [14]. Furthermore, the results of elegant experiments have provided evidence that TNAP itself may be sufficient to induce mineralization in any tissue containing fibrillar collagen [15]. Later, other factors were also identified and shown to be involved in the mineralization process. For instance, fetuin protein identified in serum that limits the production of calcium phosphate crystals and their deposition in the collagen fibrils [16]. In addition to the role of MVs as TNAP carriers during mineralization, it is also believed that these extracellular organelles are able to accumulate calcium and phosphate ions, which result in the precipitation of calcium phosphate crystals, and that these crystals, by a still unknown mechanism, are transferred from MVs into ECM.

Phosphate homeostasis maintained by the gut, bone and kidney is regulated by many hormones such as the classical ones, parathyroid hormone (PTH) and 1α,25-dihydroxyvitamin D_3_ (1α,25-(OH)_2_D_3_), as well as the fibroblast growth factor 23 (FGF23) which was recently reported to have a role in phosphate homeostasis. FGF23—a circulating hormone synthesized by osteocytes and osteoblasts in bone [17]—reduces serum phosphate and 1,25-dihydroxyvitamin D levels by acting on kidney through some FGF receptor [18]. FGF23 null mice have soft tissue calcifications, severe growth retardation, abnormalities of bone mineralization, a markedly shortened lifespan, and abnormalities of glucose metabolism [19,20].

### 1.2. Ectopic Calcifications and Defective Mineralizations

The physiological mechanisms of mineralization described above are likely to take place also in tissues other than bones and teeth under specific pathological conditions. Vascular calcification for instance in arteries of patients with renal failure, diabetes mellitus or atherosclerosis recapitulates the mechanisms of bone formation [11,21–24]. Generalized artery calcification of infancy, a rare but severe autosomal recessive disorder characterized by calcification and stenosis of arteries [25], calcification in cartilage (osteoarthritis (OA)) [26] tendons and/or in ligaments (calcific tendonitis or ankylosing spondylitis) [27] result from pathologic HA deposition in soft tissues. In patients with end-stage renal disease, FGF23 may exert a toxic effect on the cardiovascular system in a Klotho-independent manner [18]. Elevated levels of FGF23 have been associated with risks of end-stage renal disease, cardiovascular disease and mortality [28]. On the other hand, disorders that are caused by high circulating level of FGF23 are associated with hypophosphatemia while those corresponding to low circulating levels of FGF23 are associated with hyperphosphemia [20]. Heterotopic ossification is a pathological condition where bone formation occurs in extra skeletal tissues (skin, soft tissues, muscle). The two known genetic forms of heterotopic ossification are fibrodysplasia ossificans progressiva and progressive osseous heteroplasia. Fibrodysplasia ossificans progressiva is a rare heritable disorder of connective tissue disease characterized by congenital malformations of the great toes [29] and recurrent episodes of painful soft-tissue swelling that lead to heterotopic ossification [30]. Fibrodysplasia ossificans progressiva is associated with overexpression of bone morphogenetic protein 4 [31,32]. Progressive osseous heteroplasia is a developmental disorder of mesenchymal differentiation characterized by dermal ossification during infancy and by progressive heterotopic ossification of cutaneous, subcutaneous, and deep connective tissue during childhood [33]. In contrast, other diseases such as hypophosphatasia [34,35], osteoporosis [36] and rheumatoid arthritis (RA) [37] result from defective bone mineralization. Hypophosphatasia is a rare inherited disorder caused by mutations in the gene-encoding TNAP that cause a decrease in enzyme activity leading to defective bone and teeth mineralizations [34,35]. Osteoporosis is a skeletal disease characterized by low bone mass and microarchitectural deterioration resulting in bone fragility and in increasing number of bone fractures [36]. RA is characterized by inflammation in the synovium and symmetric polyarthritis. Infiltrations of the synovial tissues by inflammatory cells such as macrophages and T cell occur. Following this, local cellular proliferation of synoviocytes results in an expansion of the synovium—so called pannus—which invades and destroys articular structures [38,39]. Cytokine- and cell contact- activation of synoviocytes and monocytic cells occurs and for some of them differentiate into bone-resorbing osteoclasts [37]. Therefore, understanding the mechanisms of mineralization and bone resorption is important not only in the context of bone formation and modelling, but also in the field of calcific diseases, affecting skeletal and no-skeletal tissues [40]. Given that vascular calcification significantly increases the mortality risk in patients with chronic kidney disease [41] as well as in the general population [42], deciphering the molecular mechanisms of mineralization deserves broad consideration.

### 1.3. Matrix Vesicles and Early Stages of Mineralization

As it has been already mentioned, early stages of mineralization occur in MVs [11]. These organelles are enriched in phosphatases, especially TNAP [43–45] which can hydrolyze any phosphoesters including PP_i_, phosphatase orphan 1 (PHOSPHO1) [46,47] which can hydrolyze phosphocholine (PChol) or phosphoethanol producing P_i_. MVs also house various ion-motive ATPases [48] providing P_i_, as well as progressive ankylosis protein [45], a PP_i_ transporter from the lumen of MVs or from cells to the extracellular medium, and ectonucleotide pyrophosphatase phosphodiesterase 1 (NPP1) [45,49], which produce PP_i_ from ATP or UTP. The P_i_ pool generated by TNAP, PHOSPHO1 and ATPases significantly affects the phosphate homeostasis and is indispensable for tissue mineralization. On the other hand, the PP_i_ pool produced by ankylosis protein and NPP1 is inhibitory for mineralization [14]. Analysis of lipid composition of growth plate cartilage [50] and MVs [50–52] revealed significant phospholipase activity, although none of these enzymes have been isolated [53]. Other protein constituents of MVs and their possible roles in mineralization have been reviewed elsewhere [53,54].

### 1.4. Dietary Lipids and Bone Health

The fat and bone connection plays an important role in the pathophysiology of age-related bone loss [55]. Several reviews reported that dietary lipids such as α-linolenic acid [56], conjugated linoleic acid [57], n-3 fatty acid [58–60] could promote bone health. Long-chain n-3 polyunsaturated fatty acids (PUFA) such as eicosapentaenoic acid and docosahexaenoic acid are beneficial for bone health. They can increase bone formation, affect peak bone mass in adolescents and reduce bone loss [61]. Such beneficial effects may include the prevention or reduction of RA [57] and of osteoporosis [59,62]. However, bone mineral density is negatively associated with saturated fat intake, and men may be particularly vulnerable to these effects [63]. On the other hand, the nature or type of the diet fat were not assessed and may possibly mask the beneficial effects of some PUFAs. Nevertheless, chronic exposure to free fatty acids can be deleterious to some cell types and may contribute to lipotoxicity [64] and lead to cardiomyopathy, hepatohepatitis and diabetes [65]. Diets containing foods naturally rich in antioxidants and n-3 PUFAs could be used to treat patients with inflammatory periodontitis. However, the effect of nutritional approaches to periodontal management still need to be evaluated [66]. Skeletal lipidomics is just emerging and targeted lipidomics have not been applied to bone tissue. A partial profile of endocannabinoids and endocannabinoid-like compounds has demonstrated the presence of several long-chain fatty acid amides, some of which display potent effects on osteoblasts and osteoclasts [67]. Therefore, it became clear that an understanding of the role of phospholipases, which produce various lipids, including fatty acids, would provide additional insights into the physiological and pathological mechanisms of mineralization leading to calcification.

### 1.5. Groups of Phospholipases and Possible Roles during Mineralization

**Figure 2:**
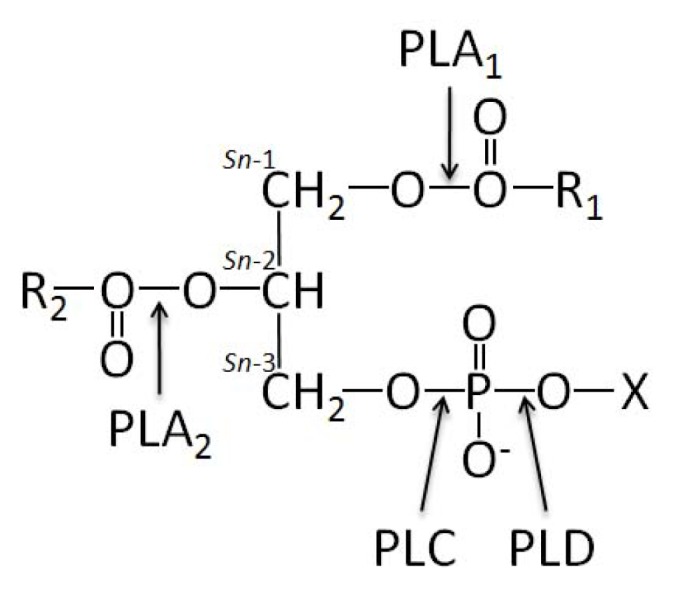
Glycerophospholipid structure and the site of action of phospholipases. The glycerophospholipid molecule consists of a glycerol-3-phosphate esterified at its *sn*-1 and *sn*-2 positions to non-polar fatty acids (R1 and R2, respectively) and, at its phosphoryl group to a polar head group, containing alcohol, X. Arrows indicate the sites of phospholipase-catalyzed hydrolysis. The carbon atoms of the glycerol backbone of the glycerophospholipid are indicated according to the stereochemical numbering (*sn*-1, *sn*-2 and *sn*-3).

There are two families of phospholipase A (PLA), PLA_1_[68,69] and PLA_2_[70–74] that hydrolyze the acyl group attached to the *sn-*1 and *sn-*2 positions of glycerophospholipids, respectively. In both cases, free fatty acids as well as lysophospholipids (LPLs) are liberated (Figure 2).

**Figure 3:**
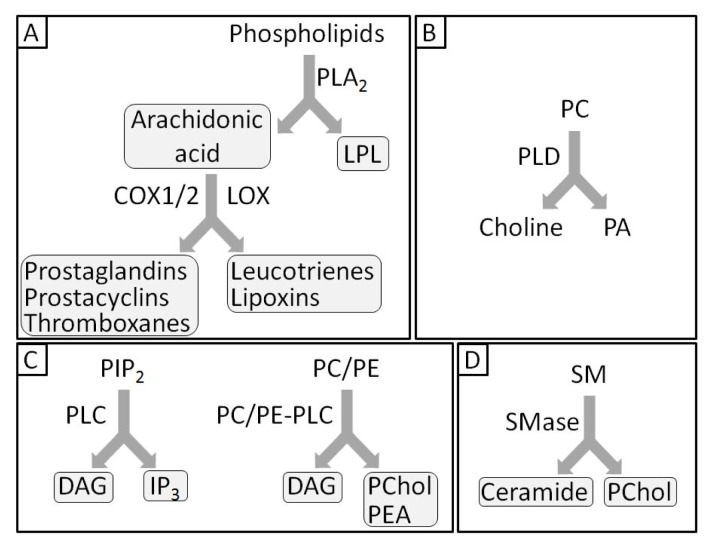
Phospholipase metabolites with biological activity at various levels of mineralization process. (**A**) Production of eicosanoids by phospholipase A_2_ (PLA_2_); (**B**) phospholipase D (PLD)-catalyzed hydrolysis of phosphatidylcholine (PC) to phosphatidic acid (PA) and Choline; (**C**) phosphatidylinositol-specific phospholipase C (PI-PLC)-catalyzed hydrolysis of PIP_2_ generating membrane-associated second messengers (inositol 1,4,5-trisphosphate (IP3) and diacylglycerol (DAG)). PC-PLC hydrolyzes PC to DAG and phosphocholine (PChol). PE-PC hydrolyzes phosphatidylethanolamine (PE) to DAG and phosphoethanolamine (PEA); (**D**) sphingomyelinase (SMase)-catalyzed hydrolysis of sphingomyelin (SM) to ceramide and PChol.

**Figure 4:**
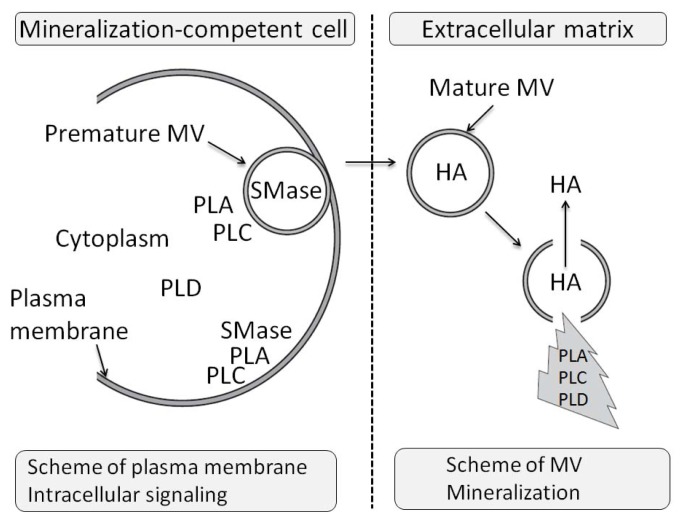
Initial steps of mineralization in which particular phospholipases can be involved.

Phospholipase C (PLC) cleaves the polar head phosphate from glycerophospholipids, producing diacylglycerol (DAG) [75,76], while phospholipase D (PLD) catalyzes the hydrolysis of the terminal phosphodiester bond of membrane glycerophospholipids, producing phosphatidic acid (PA) and free polar head group (Figure 2) [77,78]. Before discussing the potential roles of phospholipases during mineralization, it is necessary to discuss the general basis of their actions and their consequences during mineralization. Phospholipases are not only localized on cellular or organelle membranes but they can be secreted or reside in the cytoplasm. The secreted phospholipases can regulate in an autocrine or paracrine manner the osseous cells, osteoclasts and chondrocytes. Their catalytic products (Figure 3) can be involved in lipid-mediated signaling, in membrane remodelling, in endocytosis or in exocytosis of MVs. In addition, phospholipids contain phosphate, a precursor of HA formation. Their hydrolytic products may serve as a phosphate reservoir to sustain mineralization in MVs. There are at least two distinct types of targets for these lipolytic enzymes, namely those in mineralization competent cells and those in MVs (Figure 4). One can suppose that in a cell, the effects of phospholipase activities shall initiate membrane modelling, intracellular signaling events and exocytosis of MVs. In MVs, phospholipases shall break the membranous structure facilitating the release of HA crystals into ECM [11,79]. In addtion PLC and SMase will provide PChol or phosphoethanolamine (PEA) which are phosphate precursors, that could be further hydrolysed by phosphatase such as PHOSPHO1 to yield P_i_[46,47]. To illustrate the potential roles of phospholipases in MVs, it is worth looking into the lipid comparison of MVs and chondrocyte membrane fractions, which reveals small but significant differences [53]. Among them one may notice an enrichment of MV membrane in phosphatidylserine (PS) (2.3–3.5 fold), sphingomyelin (SM) (1.9–2.8 fold) and total LPLs (1.3–3.6 fold), with concomitant depletion in phosphatidylcholine (PC) content (0.8–0.9 fold) in comparison to the membrane fractions isolated from proliferating chondrocytes ([53] and Table 1). An enrichment in SM but not in LPLs was observed in membrane fractions isolated from hypertrophic cells as compared with proliferating cells (Table 1) suggesting that SMases may be silent during hypertrophy, a phenomenon that precedes MV formation [53]. This suggests that the differences in lipid compositions in membrane MVs and in plasma membranes could be not fortuitous but may have a functional significance.

## 2. Phospholipases A_1_

### 2.1. Groups, Subgroups and Specificity

There are at least nine known PLA_1_ molecules in mammals; at least six are extracellular enzymes— belonging to the pancreatic lipase gene family—and the other three are intracellular enzymes [69] (Table 2). The extracellular PLA_1_ comprise PS-specific PLA_1_ (PS-PLA_1_) [80], membrane-associated PA-selective PLA_1_ (mPA-PLA_1_α and mPA-PLA_1_β) [81,82], hepatic lipase, endothelial lipase and pancreatic lipase-related protein 2. PS-PLA_1_ is specific to PS and gives a rise to lyso-PS (LPS), while mPA-PLA_1_α and mPA-PLA_1_β are specific to PA and form lyso-PAs (LPAs). Hepatic lipase, endothelial lipase and pancreatic lipase-related protein 2, in addition to PLA_1_ activity, can hydrolyze triacylglycerols [69,83,84]. In mammals, there are three intracellular PLA_1_, a PA-preferential PLA_1_, (iPLA_1_α) [85–87]; a p125 (iPLA_1_β) [88] and KIAA0725 (iPLA_1_γ) [89] (Table 2). The physiological functions of PLA_1_ remain largely unknown in bone cells and chondrocytes in contrast to those of PLA_2_ and other phospholipases [69].

## 3. Phospholipases A_2_

### 3.1. Groups, Subgroups and Specificity

To date there are more than 30 enzymes identified in mammals that possess PLA_2_ or related activity [90,91] (Table 3). There are six types of PLA_2_: the secreted small molecular weight extracellular enzymes (sPLA_2_s) [90,92–96]; the larger cytosolic Ca^2+^-dependent enzymes (cPLA_2_s) [97–102]; the Ca^2+^-independent enzymes (iPLA_2_s) [103–107], the platelet-activating factor (PAF) acetylhydrolases (PAF-AH) [108–116]; the lysosomal PLA_2_ (LPLA_2_) [117] and the adipose-tissue PLA_2_ (AdPLA_2_) [118,119] (Table 3). Among the subgroups of secreted PLA_2_ (sPLA_2_): IB, IIA, IID, IIE, IIF, III, V, XIIA, XIIB are of human origin. Among them, the group II subfamily (IIA, IID, IIE and V) is thought to play a role in the production of several lipid mediators especially in the delayed phase of the cell activation process, because their expression levels are up-regulated under various inflammatory conditions. In contrast, sPLA_2_-IB has long been thought to be a digestive enzyme, given its abundance in the pancreas. However, the discovery of the PLA_2_ receptor (PLA_2_R) which can bind sPLA_2-_IB suggests that IB sPLA_2_ could exerts various biological responses in addition to its digestive function [120]. sPLA_2_-IA is found in cobras and kraits, -IIB is evidenced in the Gaboon viper and –IX originates from the snail venom. sPLA_2_-IIC is found in rat/murine testis. sPLA_2_-XIA and -XIB are evidenced in green rice shoots. sPLA_2_-XIII has been evidenced in parvovirus and XIV was found in symbiotis fungus and bacteria [72]. Among the cPLA_2_ -subgroups, cPLA_2_α, cPLA_2_β, cPLA_2_γ are from human origin while the three others—cPLA_2_δ, cPLA_2_ɛ, cPLA_2_η—are of murine origin (Table 3). All the six calcium independent PLA_2_—group VI iPLA_2_—have been identified in humans (iPLA_2_ A,B,C,D,E,F) as well as all the PAF PLA_2_-VIIA, -VIIB, -VIIIA and -VIIIB (Table 3). Among PLA_2_s, so far only sPLA_2_-II, -V and -X as well as cPLA-IVA [91] and iPLA_2_β [121] have been evidenced to be involved in osseous diseases. Since AA is a precursor of prostaglandins, prostacyclins and thromboxanes, as well as leukotrienes and lipoxins (Figure 3A), PLA_2_ especially cPLA_2_-α is involved in cellular signaling affecting bone formation and resorption. cPLA_2_-α is constitutively expressed in most tissues although its level of expression can be increased in response to growth factors and proinflammatory cytokines. cPLA_2_-α is unique among the PLA_2_ enzymes in having a preference for phospholipids with arachidonic acid (AA) at the *sn-2* position [122].

### 3.2. Presence of PLA_2_s in Chondrocytes and Possible Roles

Experimental evidence of the presence of specific types of PLA_2_s such as sPLA_2_-IIA, sPLA_2_-V and sPLA_2_-X, cPLA-IVA and iPLA_2_β in chondrocytes arise from analysis of human synovial fluid, especially from RA or OA patients or from the effects of cell stimulation with interleukin-1 (IL-1) and tumor necrosis factor (TNF). sPLA_2_-IIA was found in human synovial fluid of arthritic knee [123,124]. PLA_2_s were evidenced in chondrocytes stimulated with IL-1 [125]. Indeed, the expression of sPLA_2_-IIA and -V is cytokine-dependent [124]. Immunohistochemistry of RA sections revealed that sPLA_2_-IIA was generally located in synovial lining and sublining cells and cartilage chondrocytes [124]. In healthy and in OA patients, sPLA_2_-IIA is predominantly located in blood vessel endothelium and in vascular smooth muscle [126]. sPLA_2_ in the inflammation joint may originate from chondrocytes [127–130]. Indeed, cultured chondrocytes synthetise and release sPLA_2_[131]. IL-1 and TNF can stimulate the expression of mRNA encoding sPLA_2_ in chondrocytes [132,133]. IL-1 induces the secretion of PLA_2_ from chrondrocytes [134–137]. IL-1 and TNF can activate *sPLA_2_* gene expression not only in chondrocytes, but also in fibroblasts, smooth muscle cells and endothelial cells [126,138]. sPLA_2_-IIA, -IID, -V as well as cPLA_2_-IVA expressions were upregulated in human-OA chondrocytes upon IL-1, TNF, IL-6 or IL-8 stimulations [139]. Usually, among the members of cPLA_2_-IVA (Table 3), cPLA_2_-α is the most ubiquitously expressed enzyme [102].

### 3.3 Presence of PLA_2_s in Osteoblasts and Possible Roles

Inflammatory processes are characterized by increased levels of extracellular PLA_2_, IL-1 and TNF. Stimulated Fetal rat calvarial bone forming cells, treated with recombinant human IL-1 and TNF stimulated extracellular sPLA_2_[140] and the PLA_2_ activity in osteosarcoma cell lines is stimulated [141]. Fetal rat calvaria osteoblastic cells are emblematic since they continuously synthesize and release sPLA. 1α,25-(OH)_2_D_3_)—a regulator of bone biology—stimulates PLA_2_ activity in three osteoblastic cell lines: ROS 17/2.8 cells, MC-3T3-E1 cells, and MG-63 cells. 1α,25-(OH)_2_D_3_-dependent alkaline phosphatase and PLA_2_ activities were correlated with production of prostaglandin E_1_ (PGE_1_) and prostaglandin E_2_ (PGE_2_) in the MC-3T3-E1 cells [142]. PLA_2_ inhibitors (such as quinacrine or mepacrine) [143–145] and PLA_2_ activators (such as melittin) [146,147] served to evaluate PLA_2_ in osteoblasts. In MC3T3-E1 cells, quinacrine showed partial inhibitory effect on prostaglandin F_2_ (PGF_2_) induced AA release [143] while it suppressed the thrombin-induced AA release [145]. Mepacrine, significantly inhibited the bradykinin-induced AA release [144] suggesting the presence of PLA_2_ in osteoblasts. Microtubule depolymerizing agents inhibit the expression and release of sPLA_2_ by fetal rat calvarial osteoblasts [148]. MC3T3-E1 cells originating from IIA sPLA_2_-deficient C57BL/6J mouse had delayed PGE_2_ generation but introduction of type IIA sPLA_2_ augmented PGE_2_ production. This was accompanied by increased expression of both cPLA_2_ and cyclooxygenase-2 (COX-2) [149] revealing a particular cross-talk between the two PLA_2_ enzymes and COX-2. sPLA_2_ augments cPLA_2_ and COX-2 expression in mouse osteoblasts via endogenous PGE_1_[150]. IL-1α treatment induced an augmentation of PGE_2_ production by mineralizing osteoblasts involving cPLA_2_, sPLA_2_, COX-2 and PGE synthase activities [151]. However, the crosstalk between sPLA_2_ and cPLA_2_ may not hold in other cellular responses. In mouse osteoblastic cells, cPLA_2_ mRNA and protein were constitutively expressed and increased approximately 2-fold by IL-1α treatment, but secretory sPLA_2_ mRNA was not detected [152]. Using arachidonoyltrifluoromethyl ketone—a cPLA_2_ inhibitor—it was found that Cd increased cPLA_2_ activity followed by COX-2 induction, which resulted in PGE_2_ production in primary mouse osteoblastic cells [153,154]. However, the results obtained with arachidonoyltrifluoromethyl ketone should be analyzed with some caution since it is not a selective cPLA_2_ inhibitor and it may inhibit other enzymes, such as COX [155]. So far, most of the reports were focused on sPLA_2_ (among them IIA sPLA_2_) and a few were concentrated on cPLA_2_. Only recently, the presence of other PLA_2_ types in osteoblasts was evidenced. MC3T3-E1 cells possess high levels of native PLA_2_R and sPLA_2_-X is one of its high-affinity ligands. PLA_2_-VIA or iPLA_2_β is expressed in normal bone. It was suggested that iPLA_2_β mRNA is more abundant in bone forming osteoblast cells than in osteoclast cells [121]. From the findings based on knockout mice lacking iPLA_2_β, an unrecognized role of iPLA_2_β in bone formation is yet to be found. The absence of iPLA_2_β causes abnormalities in osteoblast function and BM stromal cells differentiation [121].

### 3.4. Presence of PLA_2_s in osteoclasts and Possible Roles

**Figure 5:**
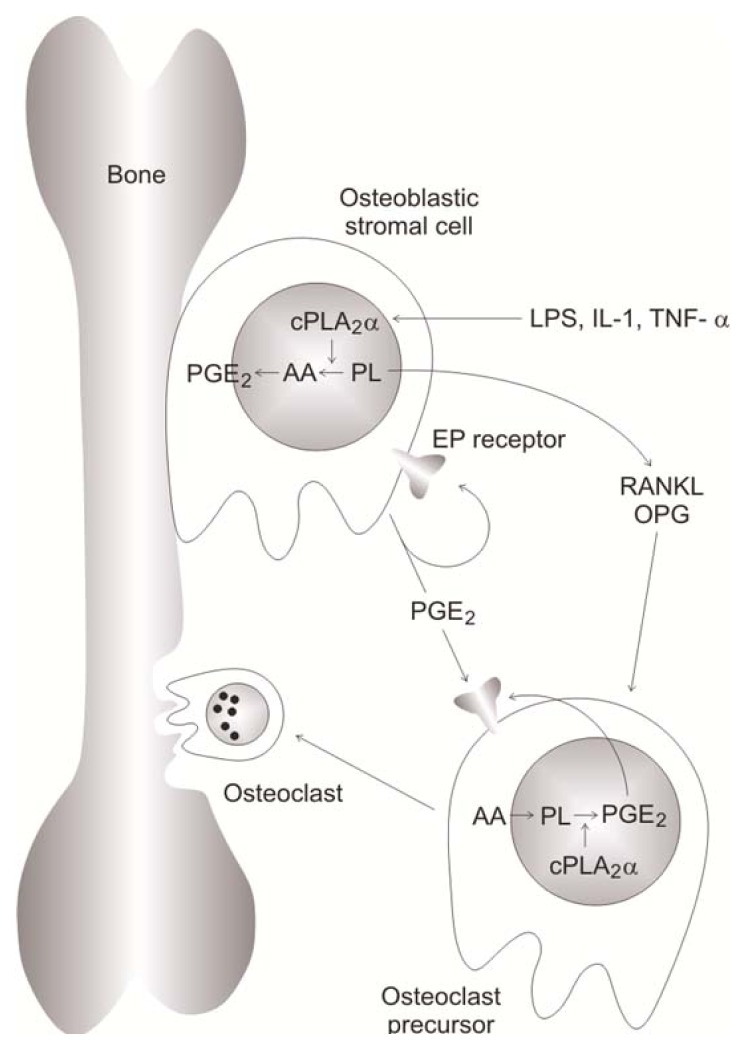
Possible role of cPLA_2_α in bone resorption. In response to lysophosphatidylserine (LPS), interleukin (IL)-1 or tumor necrosis factor-α (TNF-α), cPLA_2_α is activated and releases arachidonic acid (AA). AA is then transformed into prostaglandin E2 (PGE2) which may bind in an autocrine manner to a PGE receptor on the stromal cell or, in a paracrine manner, on the osteoclast precursor cell. Then osteoclasts derived from osteoclast precursor cells undertake bone resorption. Adapted from [122] (PL, phospholipid).

In BM cultures, IL-1 stimulated PGE_2_ production and osteoclast formation in cells from wild-type mice but not from those taken from *cPLA_2_α*^−/−^ mice [156] indicating that cPLA_2_-α is essential for PGE_2_ production. PGE_2_ may act to enhance osteoclast formation and action (Figure 5). In response to IL-1 (or other agents such as TNF-α), cPLA_2_-α is activated and PGE2 is produced and secreted. PGE_2_ may act in autocrine manner modulating the stromal cell response or in a paracrine manner on the osteoclast precursor cells. Following this, the newly formed osteoclast activates bone resorbtion [122] (Figure 5). The Enzymatic activity of cytosolic PLA_2_ was detected in human osteoclasts extracted from human fetuses and in human osteoclast-like cells differentiated from peripheral blood mononuclear cells [157]. Human osteoclasts actively produced prostaglandin, and the COX-1 pathway was implicated in the control of bone resorption. COX-2 and sPLA_2_-IIA are also implicated in osteoclastogenesis as suggested by the results obtained with the use of DFU—an inhibitor of COX-2—and KH064—an inhibitor of sPLA_2_-IIA—in ovariectomized Wistar rats. KH064 suppressed increases in osteoclast surface induced by ovariectomy while the effect of COX-2 inhibition was less marked [158].

### 3.5. Presence of PLA_2_s in Smooth Muscle Cells and Possible Roles

PLA_2_ was evidenced in vascular smooth muscle cells [159]. The isoenzyme sPLA_2_-IIA has been localized in smooth muscle cell [160–164] and has a close spatial relationship with collagen fibers [161].

### 3.6. The Expressions of PLA_2_s under Pathological Conditions

sPLA_2_-IIA is highly expressed in synovial fluid [123,126,165,166], in chrondrocytes [131], in the joints of patients with RA and to a lesser extent in synovium of OA patients but not in healthy patients [126] (Table 4). Increased catalytic activity of group II sPLA_2_ was observed in synovial fluid of OA patients [167,168]. Circulating sPLA_2_ activity correlates with juvenile RA activity [169]. The sPLA_2_-IIA activity in the serum of 212 RA patients was determined and appeared to be correlated with the Lansbury index, number of effusions, number of damaged joints, erythrocyte sedimentation rate, platelet count and low hemoglobin [170]. However, the enzyme activity is not always correlated with the severity of the RA disease [171]. sPLA_2_-IIA, sPLA_2_-IID, sPLA_2_-IIE sPLA_2_-V are more often detected in active RA than in inactive RA synovial tissues, while sPLA_2_-X is diversely expressed in both active and inactive RA tissues [124] (Table 4). This suggests that transcriptional regulation of the groups -V and -X as well as group II subfamilies are distinct. Exogenous addition of sPLA_2_-IIA, dose-dependently amplified TNF-α stimulated PGE_2_ production accompanied by increased expression of COX-2 and cPLA_2_-IIA in cultured synovial cells [172]. Exogenous addition of *Crotalus adamantus* sPLA_2_-II, as well as continuous exposure to IL-1α inhibited mineralization of the osteoid formed by fetal rat calvaria cells [173]. In normal heart, sPLA_2_-IIA was detected in coronary vascular smooth muscle cells (VSMCs) and sPLA_2_-V in cardiomyocytes beneath the endocardium. In infarcted hearts, expression of sPLA_2_-IIA and sPLA_2_-V increased in damaged cardiomyocytes and VSMCs. Expression of sPLA_2_-IID and -IIE, which were indetectable in normal heart, was elevated in damaged cardiomyocytes and VSMCs, respectively [164] (Table 4).

### 3.7. Transgenic Knockout Animal for PLA_2_ Enzymes as Models for Bone Formation and Mineralization Diseases

There are at least five knockout mice for for sPLA_2_ isoforms (-IB, -IIA, -III, -V and -X) [74,90,95,175] as well as one for the cPLA_2_α isoform [74,176–178] and at least two for iPLA_2_ isoforms -β [179,180] and -γ [181–183]. The *PLA_2_γ2a* gene coding for sPLA_2_-IIA has been knocked out in BALB/c mice by breeding them with C57BL/6 mice and then backcrossing with BALB/c mice [184]. The *PLA_2_γ2a*^−/−^ BALB/c mice displayed a reduced degree of arthritic inflammation in K/BxN autoantibody-induced mouse arthritis model as compared with wild-type BALB/c mice. This suggests that sPLA_2_-IIA is playing a pro-inflammatory role in this mouse arthritis model [184]. In contrast with *PLA_2_γ2a*^−/−^ BALB/c mice, the *PLA_2_γ5a*^−/−^ BALB/c mice lacking the sPLA_2_-V gene product exacerbated the K/BxN autoantibody-induced arthritis. Indeed, supplementation of sPLA_2_-V slowed down the K/BxN autoantibody-induced arthritis in *PLA_2_γ5a*^−/−^ BALB/c mice by facilitating phagocytic uptake of the immune complex by macrophages. This suggests that sPLA_2_-V has an anti-inflammatory effect, while sPLA_2_-IIA has a pro-inflammatory effect [184]. *cPLA_2_α*^−/−^ mice were characterized by reduced severity and incidence in collagen-induced arthritis (CIA) indicating that cPLA_2_α, plays a key role in the development of CIA [174]. A patient having a heterozygous mutation of cPLA_2_α (*PLA_2_γ4a*) with loss of cPLA_2_α function develops small intestinal ulcers, presented platelet dysfunction, and globally decreased eicosanoid production [185]. It has been previously reported that *cPLA_2_α*^−/−^ mice have developed intestinal ulcerative lesions [186]. Therefore, pharmacologic inhibition of the cPLA_2_α enzyme may induce non-steroidal anti-inflammatory drug (NSAID)-like-induced gastric and intestinal lesions. Knockout mouse models [176] indicated that cPLA_2_ is important for macrophage production of inflammatory mediators, fertility, and in the pathophysiology of neuronal death after transient focal cerebral ischaemia. iPLA_2_β-null mice exhibit defective spermatozoa mobility [179], pancreatic islet insulin secretion [180] and lower bone mass associated with a decrease in bone strength [121]. It was concluded that iPLA_2_β may be an important factor of bone formation and BM stromal cell differentiation [121].

### 3.8. Inhibitors of PLA_2_ as Drug Therapy

It was previously recognized that PLA_2_ may be an attractive therapeutic target since PLA_2_ inhibition may lead to suppression of prostaglandins, leukotrienes, and PAFs (Figure 3A) [187]. Sulfasalazine—widely used in the therapy of RA, spondyloarthropathies and inflammatory level diseases—inhibited extracellular release of sPLA_2_ from fetal rat calvaria osteoblasts suggesting that the anti-inflammatory activity may be related, in part, to the selective inhibition of the extracellular release of proinflammatory sPLA_2_[188]. sPLA_2_-IIA-inhibitor, LY333013, was administrated to 251 RA patients. Although 12-week treatment with LY333013 or methyl Varespladib—a prodrug that is rapidly converted *in vivo* to Varespladib was well tolerated, it did not significantly affect RA activity [189]. One explanation is that sPLA_2_-V has an anti-inflammatory effect, while sPLA_2_-IIA has a pro-inflammatory effect [184]. Since the inhibitor could block both enzymes [90], its action may cancel the beneficial effect. Alternatively, the regulation of TNF-dependent prostaglandin production by exogenous sPLA_2_-IIA does not depend on its enzymatic activity. Indeed, sPLA_2_-IIA mutant H48Q having only 1% of the sPLA_2_-IIA enzyme activity is as effective as the fully functional enzyme in up-regulating PGE_2_ production and in over inducing TNF-mediated COX-2 production [190]. sPLA_2_-IB, sPLA_2_-IIA, sPLA_2_-V and sPLA_2_-X can produce proinflammatory cytokines and chemokines independently of the hydrolytic activity [191]. Nevertheless, methyl Varespladib is in phase III trials for the treatment of cardiovascular diseases [192]. There is an evidence that sPLA_2_-IIA is involved in the development of atherosclerosis [193]. One possible mechanism of atherogenesis may rely on the ability of sPLA_2_ to hydrolyze the phospholipids on LDL particles promoting lipid accumulation and leading to enhanced macrophage uptake [91]. On the other hand, inhibitor of cPLA_2_α could serve as drug to treat human RA [194]. Oral administration of pyrroxyphene in a CIA in mice results in anti-arthritic activity probably due to inhibition of cPLA_2_α activity and subsequent reduction in eicosanoid levels as well as suppression of metalloproteinase (MMP) and COX-2 mRNA expression [194].

### 3.9. Effects Mediated by Arachidonic Acid and Its Pathways at Cellular Level

The fatty acid moieties that are incorporated into phospholipids vary, generating a broad range of molecular species. One of the most important fatty acids that can be released from phospholipids by PLA_2_ is AA which is converted via the COX- and lipooxygenase (LOX)-mediated pathways to eicosanoids, including prostaglandins, thromboxanes, prostacyclins, leukotrienes and lipoxins (eicosatetraenoic acid) (Figure 3A) [74,195]. sPLA_2_ does not show distinct preference for the *sn*-2 position fatty acyl chains [91]. In general, most of the sPLA_2_s have higher activity towards anionic phospholipids such as phosphatidylglycerol (PG), phosphatidylethanolamine (PE) and PS. sPLA_2_V and sPLA_2_-X can hydrolyze both PC and anionic phospholipids vesicles at comparable rates [196,197], while sPLA_2_-IA and sPLA_2_-XIV are more active against PC [91]. sPLA_2_ can release AA intracellularly prior to secretion [198], or after secretion into extracellular space. The latter is especially true for sPLA_2_-V and sPLA_2_-XV, which have high affinity for PC and act at the outer plasma membrane [199–207] or through a heparan sulfate proteoglycan (HSPG) shuttling pathway [205,207–211]. For example, sPLA_2_-IIA, -IID and -V often bind to HSPGs, internalized through caveolae/raft-dependent endocytosis, and then exert their function [205,207–211]. In addition, sPLA_2_ acts as a ligands for a M-type transmembrane sPLA_2_ receptor, which in turn activates cPLA_2_-IV [212]. cPLA_2_-IV (or cPLA_2_α) have a marked specificity for AA at the *sn*-2 position of phospholipids [97,99]. Most phospholipids such as PC, PE and phosphatidylinositol (PI) are substrates for cPLA_2_-IV. PC is a good substrate, especially radiolabeled PC, and has been often used to determine cPLA_2_-IV activity [97,98]. cPLA_2_-IV has a calcium-dependent phospholipase activity, as well as a transacylcyclase activity and a calcium-independent lysophospholipase activity [213]. Table 5 contains an overview of the overall beneficial or pathological effects of the AA pathway - including enzymes, their products and their metabolites - on bone formation or bone resorption. Most of information concerning the effects of AA metabolites comes from osteoblasts, osteoclasts and from RA chondrocytes. Several of the cytokines and growth factors implicated in the inflammatory processes in rheumatic diseases have also been demonstrated to impact osteoclast differentiation and function either directly, by acting on cells of the osteoclast-lineage, or indirectly, by acting on other cell types to modulate expression of the key osteoclastogenic factor receptor activator of nuclear factor κB ligand (RANKL) and/or its inhibitor, osteoprotegerin (OPG) [38]. In RA, synovial cells behave like osteoblasts in the induction of RANKL which is an essential ligand for differentiation of bone-resorbing osteoclasts from macrophage precursors [38,214]. It was proposed that by targeting the pathways involved in osteoclast differentiation and function, focal articular bone erosion may be attenuated in the setting of inflammatory arthritis [38].

#### 3.9.1. Effects Mediated by PGE_2_

RA synovial cells have high COX-2 and microsomal prostaglandin E synthase-1 expression suggesting that both enzymes are mediatiators of PGE_2_ production in RA inflamed joints [38]. Mice deficient in COX-2 were protected from CIA [215] while selective inhibitors to COX-2 significantly reduced the severity of arthritis in murine CIA [216]. Mice deficient in microsomal prostaglandin E synthase also exhibited significant reduction in CIA [217] or collagen antibody-induced arthritis inflammation and joint destruction [218]. On the other hand, numerous data support the notion that COX enzymes play an important role in bone formation. COX-1 can be considered as a housekeeping enzyme and is present in healthy tissues, while COX-2 is activated by tissue damage. Both enzymes have a similar Km and Vmax values for their reaction with AA [219]. NSAIDs, ibuprofen [220,221] or indomethacin [220–223] that inhibit COX-1 and COX-2 activity have been reported to inhibit fracture healing in animals (Table 6). NSAIDS can decrease heterotopic calcification in humans [224–226]. Indomethacin was shown to decrease spinal fusion rate in an animal model [227], while keterolac significantly reduces the rate of spinal fusion in humans [228]. In cultured mouse cells, indomethacin decreased TNAP activity and mineralization without significantly affecting Runx2, collagen type I and osteocalcin levels [229]. In the skeleton, prostaglandins (Figure 3A), mainly PGE_2_ produced by osteoblasts under COX-2 stimulation, play either a stimulatory or an inhibitory role in bone metabolism, depending on the physiological or pathological conditions. PGE_2_ mediates anti-inflammatory [230] as well as inflammatory effects [38,231,232]. Prostaglandins are potent multifunctional regulators in bone [233] having both stimulatory and inhibitory effects in bone metabolism [234,235]. Under physiological conditions, prostaglandins can stimulate bone formation by increasing proliferation and differentiation of osteoblasts [236]. iPLA_2_β^−/−^ null mice showed an age-related bone loss that was not accompanied by an increase in osteoclast abundance/activity. However, it was linked to increased adipogenesis from BM stromal cells and decreased osteoblastogenesis associated with higher PPARγ and lower Runx2 levels [121]. These findings suggest that iPLA_2_β is involved in bone formation and BM stromal cells differentiation. On the other hand, under pathological conditions like osteoporosis, RA, OA and periodontis, prostaglandins can activate bone resorption by increasing the activity of osteoclasts [237,238]. PGE_2_ is detected at high level in the synovial fluid of RA patients [239,240]. PGE_2_ mediates pain and inflammatory responses [38,231,235]. Indeed COX-2 inhibitors are effective for decreasing pain in RA [231,241]. cPLA_2_ in osteoblasts, upon stimulation by IL-1, TNFα or lipopolysaccharide, produces PGE_2_ which may acts through one or more receptors in autocrine manner as well as in a paracrine manner on the osteoclast precursor cells. Then the number of osteoclast cells increases leading to bone resorption (Figure 5). Results obtained using MG63 osteoblast-like cells cultured on commercially pure titanium surfaces of two different roughnesses in the presence of media containing 1α,25-(OH)_2_D_3_ without or with quinacrine (a PLA_2_ inhibitor) indicated that surface roughness and 1α,25-(OH)_2_D_3_ transmit their effects through PLA_2_, which catalyzes one of the rate-limiting steps in PGE_2_ production [242]. PGE_2_ exerts indirect effects on osteoclastic differentiation through osteoblasts but can have direct effects on osteoclast precursor cells and mature osteoclasts [122,243]. A cPLA_2_α-deficient mice had reduced CIA symptoms which suggests that AA, as a precursor of prostaglandins (Figure 3A) and leukotrienes is the inflammatory mediator in the development of CIA [174]. Indeed, it was suggested that cPLA_2_ antagonists might be effective in reducing inflammatory bone resorption associated with RA and periodontal diseases [122]. Therefore, analysis of AA metabolites should become an obvious target for further investigations with potential pathophysiological, therapeutic and regenerative outcomes [244]. Beneficial effects of n-3 PUFAs and conjugated linoleic acids on bone formation rate are likely due the modulation of COX-2 mediated biosynthesis of PGE_2_[244]. It was proposed that increased consumption of foods providing n-3 PUFA and conjugated linoleic acid will balance eicosanoid biosynthesis by decreasing pro-inflammatory AA concentration and will help to prevent or relieve diseases associated with increased n-6 fatty acid derived eicosanoid production [244].

#### 3.9.2. Effects Mediated by PGF_2_α and PGD_2_

Other prostanoids and leukotrienes may also play a role in bone metabolism and bone remodeling but they are less defined. Prostaglandin F_2_α (PGF_2_α) contributes to osteoblast functions. Locally produced PGF_2_α might be beneficial in promoting osteogenic differentiation of adipose tissue-derived mesenchymal stem cells [245]. It has been reported [246] that PGF_2_ stimulates Na_+_-dependant phosphate transport (P_i_ transport) activity [247], playing important role in the mineralization of osteoblast-like cells. 15-deoxy-Δ^12,14^-prostaglandin J_2_, a prostaglandin D_2_ (PGD_2_) metabolite, by binding to and activating PPARγ, may also modulate TNAP expression and mineralization [248]. PGD_2_ has a stimulatory activity on osteoblast calcification [249]. In addition to COX-metabolized prostaglandins, LOX-mediated generation of leukotrien B4 may be involved in TNAP post-translational activation during osteoblastic differentiation [250].

### 3.10. Effects Mediated by Lysophospholipids and Their Pathways at Cellular Level

**Figure 6:**
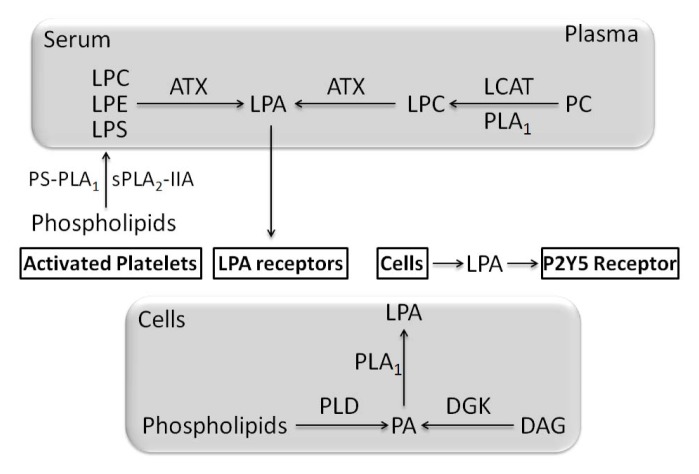
Production of lysophosphatidic acid (LPA) via two main pathways: Half of serum LPA is formed through the generation of lysophospholipids (LPLs), such as lyso-PC (LPC), lyso-PE (LPE), and LPS, by secreted PLA_2_ (sPLA_2_-IIA) or PS-PLA_1_ from membrane phospholipids of activated platelets, followed by conversion of the LPLs to LPA by autotaxin (ATX). The other half of serum LPA can be generated by sequential action of lecithin: cholesterol acyltransferase (LCAT) or PLA_1_ and ATX. LPA produced by ATX has various roles mediated by LPA receptors. LPA in cells may originate from PA. In this pathway, a PLD or DAG kinase convert lipids in PA and then PA is hydrolyzed by PLA_1_ or PLA_2_.

LPLs are produced either by PLA_1_ or PLA_2_ and are potential lipid mediators [252]. Among various LPLs detected lyso-PC (LPC) is the most abundant with a plasma concentration of several hundred micromoles per liter [252]. Other LPLs such as lysophosphatidylglycerol (LPG), lyso-PE (LPE), lyso-PI (LPI) and LPS are present at low concentration [252]. GPR55—a G protein-coupled receptor and cannabinoid-sensitive receptor—is activated by 1-acyl LPI [253–256]. GPR55 has only a distant phylogenetic relationship to CB1 and CB2 cannabinoid receptors, but clusters with the LPA receptor LPA_4_[257]. Since LPI and LPA are similar, LPI is a good agonist of GPR55. The most active species is 2-arachidonyl-*sn*-glycero-3-phosphoinositol which can be considered as one of the possible natural substrates of GPR55 [258]. GPR55 expression was found to be 8-fold higher in osteoclasts than in monocytes from human healty donors. The GPR55-agonist LPI inhibited mouse osteoclast formation but it stimulated mouse and human osteoclast polarization and resorption *in vitro*[259]. Although GPR55-agonist LPI induced a decrease in osteoclast number it stimulated osteoclast function. Treatment of human osteoclasts with LPI caused activation of RhoA and ERK1/2 [259] suggesting that activation of GPR55—via inducing a signaling pathway—promotes a regulatory mechanism in osteoclasts. Male *GPR55*^−/−^ mice but not female *GPR55*^−/−^ mice presented a decrease in bone resorption and an osteopetrotic phenotype with an increasing osteoclast number [259]. Although osteoclast number increased, osteoclast function was impaired, consistent with the role of GPR55 in stimulating osteoclast function [259]. *GPR55*^−^*/*^−^ mice are resistant to neuropathic and inflammatory pain suggesting that GPR55 antagonists may serve to treat arthritic pain [260]. GPR55 has the potential to influence bone resorption and could be a target to treat bone diseases, such as osteoporosis, by inhibitory drugs, or calcification diseases (osteopetrosis, *etc*.) by activatory drugs. The role of LPA in skeletal biology has been reviewed [261] and only selected findings will be discussed here. LPA is a stimulator of resting zone chondrocyte proliferation and maturation and an inhibitor of chondrocyte apoptosis. LPA decreases the abundance of p53 to alter p53-target gene expression resulting in the inhibition of caspase activity [262]. Different LPAs vary according to acyl chain length and degree of saturation. Typically, 1-oleoyl LPA is the specimen used in describing its effects on cell and tissues [261]. LPAs are present in plasma at around high nM to low μM concentrations [263–268] and at elevated levels at sites of tissue injury or inflammation [267]. LPA species with saturated fatty acids (16:0, 18:0) and unsaturated fatty acids (16:1, 18:1, 18:2 and 20:4) have been detected in serum, plasma, and activated platelets [269–271]. LPA is an important intermediate product of the synthetic pathway for phospholipids and triacyglycerols in many cell types from various species. In this pathway, LPA is produced by acylation of glycerol-3-phosphate. However, so far, there is no evidence of LPA release into the extracellular fluid or accumulation in plasma membrane and this pathway in not considered to be involved in extracelular LPA signaling [272]. LPA is produced both in the cells and in biological fluids, such as serum and plasma, by distinct pathways [273,274]. (1) In serum and in plasma, LPA is mainly converted from LPLs by a lyso-PLD [275]—later identified as autotaxin (ATX) [276,277]. LPL in plasma and serum are produced by PLA_1_-like enzymes [266,272] and by a lecithin: cholesterol acyltransferase [278] (Figure 6). Another source of LPLs (about half part of the total amount) in serum are activated platelets. In platelets, PS-PLA_1_[279] is involved in the production of 2-acyl-LPL while sPLA_2_-IIA could be involved in the production of 1-acyl-LPL [70,280] (Figure 6). The PS-PLA_1_ and PE substrate-preferred sPLA_2_-IIA are extracellular enzymes. Since their substrates are in the inner leaflet of the lipid bilayer, the phospholipids asymmetry should be disrupted and indeed such phenomenon is known to occur in activated platelets, as well as in apoptotic cells and cytokine-stimulated cells [272]. The exposed phospholipids could be targets for PS-PLA_1_ and sPLA_2_-IIA enzymes. LPA production by this pathway could be involved in wound healing and inflammation [272]. LPAs are not only produced and released by activated platelets [281] but also by activated inflammatory cells such as erythrocytes and white blood cells [280]. In addition LPA may originate from cancer cells, fibroblasts or adipocytes. Lipoproteins are also a source of LPA. Therefore, the concept of local (autocrine, paracrine) action of LPA which has been demonstrated in platelet aggregation should be extended to other cellular responses. Moreover, several pathological conditions such as cancer, injuries, hematoma, renal failure, are associated with a relatively high production of LPA, thus constituting an interesting marker of cell aggression [282]. In contrast to LPA in plasma and serum, LPA in cells may originate from PA [273]. In this pathway, a PLD or DAG kinase convert lipids in PA and then PA is hydrolyzed by PLA_1_ or PLA_2_. There was no direct evidence of LPA production from PA by PLA_2_ (such as the secretory type or cytosolic type), however a PA-selective PLA_1_ (PA-PLA_1_a/LIPH or mPA-PLA_1_a) could be involved in the production of LPA [81]. As reviewed [273], very little is known about this pathway. For example, production of LPA by mPA-PLA_1_a has been evidenced in hair follicles. Then, the secreted LPA can activate the P2Y5 receptor which is the closest homolog of LPA_4_, a G-protein-coupled receptors (GPCR) for LPAs [283] (Figure 6). Albumin binds with high affinity to LPAs and may serve as LPA carrier [284]. Synovial fluid of patients with RA contains a significant amount of LPA. To evaluate its effects, the synovial fluid from RA patients was used in fibroblast-like synovial cells and was found to stimulate COX-2 induction in these cells [285]. Lyso-PLD, LPC and LPA-producing enzymes are present in synovial fluid [285]. Platelets can contribute to LPA production in bone tissue [286,287]. LPA biosynthesis can occur in response to purinergic signaling via P2X7 receptors [288,289]. There are distinct mechanisms involved in the control of the paracrine secretion of LPA [280,286,290]. LPA can affect neighbouring cells in an autocrine/paracrine manner via interactions with a subset of six GPCRs for LPAs so called LPA_1_-LPA_6_[291]. Addition of LPAs stimulated the proliferation of primary rat osteoblasts [292], osteoblast-like MC3T3-E1 cells [293], primary human osteoblasts [294], human osteosarcoma cell line G92 and MG-63 [294,295] as well as proliferation of rat primary chondrocytes [296]. LPA addition induced chemotaxis, elevated resistance to apoptosis, activated mitogen-activated protein (MAP) kinases, and elevated Ca^2+^ in osteoblasts, the precursors of osteocytes [292,294,297–299]. LPA stimulated osteoblast fibronectin assembly and binding with concomitant changes to the cytoskeleton [300–302]. It was predicted that osteocytes could be a target for LPA and indeed LPA stimulated dentrite outgrowth in MLO-Y4 osteocytes [303]. LPA induced osteogenic differentiation of human mesenchymal stem cells hMSC-TERT by interacting with LPA_1_ and LPA_2_ receptors. LPA_1_ receptor activation—coupled to a rise of Ca^2+^—promotes osteogenic differentiation while LPA_4_ receptor activation, coupled to cAMP, inhibits differentiation [304]. LPA_1_^−/−^ mice exhibits impaired suckling behavior and neurological abnormalities [305,306] as well as defects in bone formation leading to osteoporosis [306]. Since LPA_1_ and LPA_4_ displays completely opposite effects, as expected LPA_4_^−/−^ mice had, as expected, an increased bone volume, trabecular thickness and trabecular number [304]. The potential effects of LPA on osteogenesesis or osteoclasis may depend on the respective expressions of LPA receptors [306]. In osteoclasts, LPA acts through multiple receptor subtypes to elevate intracellular Ca^2+^ (Ca^2+^_i_), induce cellular retraction, activates nuclear factor of activated T cell 1 (NFAT1) and prolongs osteoclast survival [307]. Undifferentiated and differentiated ATDC5 chondroprogenitor cells were found to express LPA [308].

### 3.11. The Effects of PLA Metabolites at Matrix Vesicle Level

*In vivo*, increasing content of LPLs in the growth plate is associated with the onset of mineralization events and participates in the breakdown of MV membrane. Depletion of PC and increase in LPC are characteristic of MVs and are already observed in the microvilli from which MVs derive [309]. Indeed, 10%–15% of the total phospholipids in MVs from chicken growth plate cartilage are LPLs [52,310–312]. LPLs may destabilize the MV membrane, leading eventually to the loss of membrane integrity and release of MMP-3 into ECM, stimulating the ECM components digestion [294] as well as release of calcium phosphate crystals from MVs to ECM [50]. LPLs could also serve as a reservoir for generation of pro-mineralizing P_i_, as TNAP was reported to hydrolyse LPLs [313]. This enzyme, purified from the osseous plate, displayed broad substrate specificity. In fact, TNAP from osseous tissues as well as hyperthrophic chondrocytes is a multifunctional enzyme capable of hydrolyzing phosphate monoesters, PP_i_ and phosphodiesters [314,315]. In addition, NPPs present in MVs share the same capability to hydrolyze phosphodiester bonds, acting on distinct substrates that include nucleosides triphosphates, LPLs and choline phosphate esters [49]. The composition of lipids in chicken growth plate cartilage MVs has been described to change significantly during the process of mineralization and it is accompanied by a rise in free fatty acid and LPLs produced by PLAs identified in MVs [50]. By analysing chicken cartilage MV phospholipid content and composition, it was suggested that LPE, detected by UV absorption at 205 nm which indicates the presence of unsaturated fatty acids found in the MV membrane, must have arisen from action of PLA_1_[50]. On the other hand, PLA in MVs displayed a PLA_2_ activity, which was Ca^2+^-dependent, highly selective for intramembranous, as opposed to externally added phospholipids substrates, had optimal activity at pH 8 and hydrolyzed PC in preference of PE or other membrane phospholipids [53]. It has been observed that MVs released by hypertrophic chondrocytes contain higher levels of TNAP and PLA_2_ specific activities than MVs released by the resting zone cells [316,317]. Addition of 10^−8^ to 10^−9^ M of 1α,25-(OH)D_3_ significantly increased PLA_2_ activity in MV but not in plasma membrane.

## 4. Non-Specific Phospholipase C

### 4.1. Groups, Subgroups and Specificity

PLC cleaves the polar head phosphate from phospholipids, producing DAG (Figures 2,3C). The polar head phosphate is released into the cytoplasm, whereas DAG remains as an integral component of the membrane. The observation that certain activators of protein kinase C (PKC) function as DAG or potent tumor promoters [318] suggests the possibility that uncontrolled activation of PLC may trigger a mitogenic response. In mammalian cells, PLC has been implicated in intracellular signal transduction, vesicle transport, endocytosis, exocytosis, ion channel function, mitosis, cytoskeletal reorganization, and neuronal signal transduction [319]. On the basis of their substrate specificity, two major classes of PLC, namely PI-specific PLC (PI-PLC), with specificity towards PI and non specific PLC, PC-PLC, hydrolysing PE or PC, were identified. PC-PLC has been found in many organisms, from bacteria to mammals [319]. PC-PLC catalyzes the hydrolysis of PC, generating PChol and DAG. PC-PLC was found in the cytoplasm, plasma membrane, and the nucleus. PC-PLC is a potential target for therapy in inflammation-associated diseases such as atherosclerosis [320,321]. PC-PLC is implicated in cytokine signaling pathways, such those of interferon-γ [322], transforming growth factor-β [323] and TNF-α [324]. D609, an inhibitor of PC-PLC, blocked the progression of atherosclerotic lesions in ApoE^−/−^ mice. The lesions contained less lipid and matrix MMPs, fewer macrophages and more smooth muscle cells and collagen [325]. In the future, PC-PLC might serve as a marker in diagnosis of atherosclerosis in the future and as a new target for atherosclerosis therapy.

### 4.2. Presence of PC-PLC in Chondrocytes and in Osteoblasts and Its Possible Role

To the best of our knowledge, there are almost no reports on the presence and role of PC-PLC in chondrocytes. A pathway for the liberation of AA in osteoblasts involves the non specific hydrolysis of PI and PE by PLC followed by the deesterification of DAG. This pathway can be activated by a phorbol ester through a protein kinase C-independent mechanism [326]. Little information is available on the roles of PC-PLC in osteoblasts, especially during intracellular cell signaling in osteoblast-like MCT3T3-E1 cells. IL-6 stimulates bone resorption and induces osteoclast formation [327]. IL-6 synthesis is induced by IL-1 which is a potent resorptive agent and osteoblasts, rather than osteoclasts, have receptors for many resorptive molecules [328]. The regulatory mechanism of IL-1action in MCT3T3-E1 cells consists of activation of PKC via PC-PLC [329]. Inhibitors of PKC (staurosporine and calphostin) or of PC-PLC tricylodecan-9-yl xanthogenate (D609) enhanced the IL-6 secretion by IL-1 [329] suggesting that IL-1, by activating PKC via PC-PLC limits IL-6 synthesis, while possibly another pathway induced by IL-1 stimulates IL-6 synthesis. IL-6 synthesis is also regulated by TNF which activates PKC via PC-PLC in MCT3-E1 cells [330]. Hydrolysis of sphyngomyelin and PC are activated upon TNF stimulation [330]. PGF_2_α-induced proliferation in osteoblast-like MC3T3-E1 cells was accompanied by sustained increase in DAG which, in turn, was mediated mainly via tyrosine kinase(s)-dependent PC hydrolysis by a PC-PLC [331]. One of the mechanisms leading to lead-induced apoptosis in cultured rat primary osteoblasts may involve a PC-PLC activity [332].

### 4.3. Presence of PC-PLC in Osteoclasts and Possible Roles

A possible role of a PC-PLC in BM cells has been evidenced using a PC-PLC specific inhibitor D609. It was found that TNF-α promoted RANKL-induced osteoclastogenesis, at least partially, through the PC-PLC/inositol-1,4,5-trisphosphate (IP_3_) receptors/NFAT1 pathway [333].

### 4.4. Presence of PC-PLC in Smooth Muscle Cells and Possible Roles

IL-4 induced OPG mRNA levels and protein secretion by 5-fold in a dose- and time-dependent fashion in human coronary artery smooth muscle cells. Addition of inhibitor D609 decreased OPG expression controlled by IL-4-induced signal transducer and activator of transcription (STAT6) activation suggesting that PLC may participate in the transformation of smooth muscle cells towards an osteoblastic phenotype [334].

### 4.5. The Effect of PLC Metabolites in Matrix Vesicles

It has been described [50] that the PE and PC content of the MV membrane decreases during mineralization with some accumulation of DAG in MVs, suggesting the involvement of a non-specific PLC activity. In addition there was some accumulation of monoacylglycerol during MV mineralization indicating a lyso-PLC activity [50]. The MV membranes are rich in both PE and PC and may act as a reservoir for both phospholipid classes during early stages of mineralization. Among different enzymes involved in further metabolism of PLC metabolites (Figure 3C), PHOSPHO1, a soluble cytosolic phosphatase entrapped inside MVs [47,53,54], was found to be implicated in generation of P_i_ for mineralization [46,47]. PHOSPHO1 is capable to hydrolyse PEA and PChol to generate P_i_[46,47]. The enzyme activity is highly Mg^2+^-dependent, with optimal concentration of Mg^2+^ between 1 and 10 mM. The apparent *K*_m_ values amounted to 3.0 μM for PEA and 11.4 μM for PChol [46,47]. It has also been hypothesized that PEA is a natural substrate for TNAP since an increase in its urinary excretion in patients diagnosed with hypophosphatasia has been observed [335]. Kinetic and biochemical analysis of TNAP isolated from human Saos 2 cells revealed that this enzyme possesses also phosphatase activity towards PEA [336]. One possible role of PLC in MVs is to produce PEA and PChol which can be further hydrolyzed by PHOSPHO1 and TNAP producing P_i_ necessary for MV-mediated mineralization [53]. So far, there are no reports that point out experimental evidences of PLC activity toward PC or PE in chondrocytes [53]. Alternatively, a sphyngomyelinase activity could contribute to the production of PEA and PChol.

## 5. PI-Specific Phospholipase C

### 5.1. Groups, Subgroups and Specificity

PI hydrolysis by PLC results in the production of two second messengers, IP_3_ (Figures 2,3C) which mobilizes calcium ions from intracellular calcium stores, and DAG, a physiological activator of PKC isoforms [337]. PI-PLC is a soluble protein that is localized mainly in the cytoplasm and is translocated to the plasma membrane where it hydrolyzes PI 4,5-bisphosphate (PIP_2_) in response to cell activation [75]. PIP_2_ is a precursor not only of IP_3_ but also of PI 3,4,5-trsiphosphate (PIP_3_), which is produced by the action of PI-3 kinase. Strict regulation of the levels of PIP_2_ and PIP_3_ is very important for maintaining homeostasis of the body. PIP_2_ regulates a variety of cells functions, among them cytoskeletal rearrangement [338], membrane trafficking such as endocytosis of the EGF receptor [339], ion channel activity [340]. PIP_3_ transduces intracellular signals related to cell proliferation or motility. Therefore defects in the generation/degradation of PIP_3_ cause cancer, diabetes and inflammation [341,342]. Thirteen different mammalian PI-PLC isoforms have been described, and divided into six subclasses namely β [343–345], γ [343,346,347], δ [343], ɛ [348–351], ζ [352] and η [353–355] each of which comprising different isoenzymes: PI-PLC-β1–β4, γ1 and γ2, δ1–δ4, ɛ, ζ and η. The activation mechanisms of several PLCs have been clarified [356] (Table 7). The isoforms can be divided on the basis of amino-acid sequence and their ways of activation. All the PI-PLC isoforms contain catalytic X and Y domains. Outside of the core conserved regions, there is diversity in protein structure that reflects the range of mechanisms utilized for regulation of these enzymes. Except for PI-PLC-ζ, all PLC isoenzymes have a pleckstrin domain (PH) which binds membrane phosphoinositides or regulatory proteins [356,357]. PI-PLC β1–β4 isoenzymes are composed of subtype specific domains and conserved domains, such as catalytic core regions designated as the X and Y domains, which are located between the EF-hand motif and two phospholipid-binding regions the PH and C2 domains [356]. The catalytic core of PI-PLC-γ isozymes comprises a split PH domain flanking two tandem src homology 2 (SH2) domain inserts of the catalytic core of PLC-γ and SH3 domain between the two halves of the TIM Barrel catalytic domain [356]. Members of the PLCγ class are regulated by receptors that are coupled to tyrosine kinases [357]. PI-PLC-δ, comprising of a PH domain, EF hand motif, X and Y domains and a C2 domain is considered as the most basic isozyme due to its simple structure [356]. PI-PLCɛ is unique in relation to other PLC isoforms in terms of its ability to be regulated by multiple signaling inputs from both Rho family GTPases and heteromeric G protein. [358,359]. Two forms of PI-PLC-ɛ differing in size by 25 kDa were found and are designated as PI-PLC-ɛ1a and PI-PLC-ɛ1b [360]. No functional differences between the two splice variants have as yet been identified. The identification of an independent nuclear polyphosphoinositides signaling machinery has paved the way to find new roles for these molecules. Although several distinct isozymes of PI-PLC have been detected in the nucleus, the isoform that has been most consistently highlighted as being nuclear is PI-PLC-β1 where signaling occurs not only at the plasma membrane but also in the nucleus [356]. Indeed, all the four members of PI-PLC-β contain a high proportion of basic residues at their *C*-terminal domain, critical for nuclear localization [361,362]. Nuclear PI-PLC-β1 has been linked with either cell proliferation or cell differentiation [363]. The presence of other PI-PLC isozymes, such as PI-PLC-γ1, -δ1, -δ4, and PLC-ζ, in the nucleus have been reported [362]. PLC-γ1 is essential for cell proliferation and cell differentiation [364–367].

### 5.2. PI-PLC in Tissues

The distribution of PI-PLC isozymes is tissue and organ specific [356,359,362,369]. PI-PLC-β1 is highly expressed in the cerebral cortex and hippocampus [370] compared to limited expression of PI-PLC-β2 in hematopoietic cells [369,371]; PI-PLC-β3 is found in brain, liver, and parotid gland [372]; PI-PLC-β4 is present at the highest level in cerebellum and retina [373]. Two mammalian subtypes of PI-PLC-γ isozymes have been identified. PI-PLC-γ1 is abundantly expressed in embryonal cortical structures, neurons, oligodendrocytes and astrocytes [374]. The expression of PI-PLC-γ2 is primarily limited to cells of hematopoietic lineage. PI-PLCδ1 is present at high abundance in brain, heart, lung, skeletal muscle and testis. PI-PLC-δ3 is detected abundantly in brain, skeletal muscle and heart [375]. PI-PLC-δ4 mRNA is expressed in various tissues with the highest levels detected selectively in brain, skeletal muscle, testis and kidney [376]. PI-PLC-ɛ mRNA expression has been detected in brain, lung, and colon, with the highest expression detected in heart. PI-PLC-ζ expression within the testis is sperm-specific. Two PI-PLC-η isozymes, PI-PLC-η1 and PI-PLC-η2, were identified in humans and mice. The highest level of PI-PLC-η1 mRNA was observed in brain and kidney and smaller levels were detected in lung, spleen, intestine, thymus and pancreas [353]. As evaluated from the EST database in NCBI Unigene (http:/www.ncbi.nlm.nih.gov/sites/entrez?db=unigene), *PI-PLC-β2*, *PI-PLC-γ1*, *PI-PLC-γ2*, *PI-PLC-δ1* and *PI-PLC-ɛ* genes are expressed in bone tissues but to a limited degree compared with their expressions in other tissues [356].

### 5.3. Presence of PI-PLC in Chondrocytes and Possible Roles

Chondrocytes treated with PLC from *Clostridium welchii* divided repeatedly but failed to synthesize metachromatic matrix [377] suggesting that PLC may have a functional role in chondrocytes. Most of the experimental evidence of PI-PLC in chondrocytes comes from the use of U-73122, a PLC inhibitor. Earlier experimental evidence of PLC activity derived from articular chondrocytes upon fluid-induced shear. The shear-induced change in matrix molecule metabolism was influenced by NO synthesis, G protein activation and PLC activation [378]. The molecular mechanisms on the effects induced by mechanical stress indicate the participation of PI-PLC. Mechanical stress induced Ca^2+^ influx in primary cultures of rabbit articular chondrocytes and stimulated PI-PLC activity [379]. Periodic mechanical stress of rat chondrocytes—leading to chondrocyte area expansion and migration—implies phophorylation of tyrosine kinase protein Src, which in turn activates PI-PLC that regulates ERK1/2 activity [380]. Since the later activity was inhibited by U73122, it was assumed that a PI-PLC-γ1 was involved in this pathway [380] probably due to its activation by tyrosine protein kinase [381]. The use of calcium blockers or ionophores served to identify the presence of Ca^2+^-sensitive PI-PLC. Insulin-like growth factor-1 induced an increase in Ca^2+^_i_ that was pertussis toxin (PTX) dependent in articular chondrocytes from 21-day-old rabbits. Treatment with U-73122 [382] partially blocked the Ca^2+^_i_ increase suggesting that PLC coupled to a PTX-sensitive G protein was present in chondrocytes [383]. In HIG-82 synovial cells, the ionophore ionomycin stimulated phosphoinositide hydrolysis indicating the expression of a Ca^2+^-sensitive PI-PLC activity in these cells [384,385]. PI-PLC (very often abbreviated as PLC) activity has been evidenced during determination of signaling pathways induced by vitamin D metabolites, sex hormornes, 17β-estradiol, adrenocorticotrpin, eotaxin-1, FGF-3, *etc.* The resting zone chondrocytes from costochondral cartilage responds preferentially to the vitamin D metabolite 24*R*,25-dihydroxyvitamin D_3_ (24R,25(OH)_2_D_3_), whereas hypertrophic chondrocytes respond preferentially to 1α,25(OH)_2_D_3_[386,387]. In chondrocytes from the costochondral cartilage growth zone, 1α,25(OH_2_)D_3_ causes a rapid increase in PLA_2_ activity [388] producing AA and LPL. AA can stimulate PKC activity [389] and can serve as a substrate for COX-1. LPL activates PI-PLC (possibly PI-PLC-β) since it is a G protein sensitive (Gαq) pathway- resulting in IP_3_ and DAG production which contributes to PKCα activation and downstream activation of ERK1/2 [388,390–393]. PI-PLC-β1 and PI-PLC-β3 were proposed to be involved in LPL-activation of PI-PLC [393] but they are not expressed in bone or in BM [356]. Growth-plate chondrocytes respond to17β-estradiol in a sex-specific manner by inducing an increase in IP_3_ which suggests the involvement of PI-PLC activity [394] as reported earlier [395,396]. It was suggested that in resting zone chondrocytes, 24*R*,25(OH)_2_D_3_ was initiating LPA mediated stimulation of G-induced PI-PLC activity [397]. Resting chondrocytes treated with melanocortin peptide or/and ACTH showed elevated basal Ca^2+^ level that was decreased [398] by U-73122 [382]. The sex hormorne, testosterone, and its metabolite, 5α-dihydrotestosterone (DHT), play an important role in skeletal development in males during adolescence [399]. The effects of DHT were observed in resting-zone chondrocytes from rats in a sex-specific manner *i.e.*, only in males. PI-PLC was required for the DHT–dependent activation of PKC [400] as evidenced by the effect of U-73122. The eotaxin-dependent matrix metalloproteinase secretion in human chondrosarcoma cell line SW1353 is regulated by a PI-PLC-PKC cascade and c-Jun *N*-terminal kinase/MAP kinase pathways [401]. PI-PLC-γ mediates FGF-3-induced a STAT1 in ATDC5 chondrogenic cells [402].

### 5.4. Presence of PI-PLC in Osteoblasts

Osteoblast-like osteosarcoma UMR-106 cells possess at least two distinct PLC activities, one predominant in the cytosol and activated by increasing cytosolic Ca^2+^ with PI as the substrate. The second enzyme, a GTP-activated PI-4, 5-bisphosphate (PIP_2_)-specific PLC is found in the plasma membranes [403]. PI-PLC-β1, -β3, -γ1, -γ2, and -δ1 were detected by Western blot in osteosarcoma MG-63, MNNG/HOS, OST, U-2/OS, and SaOS-2 cell lines, while PI-PLC-β2 was only expressed in MG-63 and MNNG/HOS cells [404]. PI-PLC-β2 is involved in the mechanotransduction in primary osteoblasts [404]. PLC-γ1 plays an important role in the regulation of cell proliferation and differentiation by generation of the second messengers, DAG, and IP_3_. PLC-γ1, ERK1/2, and nuclear factor κB (NF-κB) signaling pathways are stimulated while p38 MAP kinase is inhibited by H_2_O_2_-induced oxidative stress during rabbit BM stromal cell differentiation. [405]. Elevated extracellular Ca^2+^ (Ca^2+^_e_) stimulates both chemotaxis and mitogenesis of MC3T3-E1 osteoblasts via a calcium-sensing receptor (CaR). Ca^2+^_e_-mediated chemotaxis of these bone-forming cells is dependent on PLC [406]. Alternatively, most of the evidence of the presence of PI-PLC in osteoblasts originates from ligand-induced signaling pathways involving Ca^2+^_i_ increase with the production of DAG and IP_3_ as reported below.

#### 5.4.1. Endothelin-1 Induced Signaling Pathway

Endothelin-1 (ET-1)—a vasoactive peptide derived from endothelial cells—by binding to high-affinity receptors in MC3T3-E1 osteoblast cells, induces PLC activation with the production of two second messengers, IP_3_ and DAG and a biphasic increase in Ca^2+^_i_, as measured with a fluorescent indicator, fura-2 [407,408]. It has been shown that ET-1 inhibits osteoclast bone resorption by a direct effect on cell motility and that it can also activate PLC in the osteoblast [409]. In MC3T3-E1 osteoblast-like cells, ET-1 acting through ET receptor, links to a stimulation of Pi transport via activation of PKC through both phosphoinositide and PC hydrolyses [410]. In osteoblast-like MC3T3-E1 cells, various ET peptides and their homologous sarafotoxins generate PGE_2_ release through an ET(A) receptor subtype. PLC-dependent calcium activation mechanisms seem to be involved [411].

#### 5.4.2. Basic FGF Induced Signaling Pathway

Basic FGF selectively stimulates sodium coupled Pi transport activity in osteoblast-like cells. Signaling mechanisms responsible for this effect involve mainly activation of PI-PLC-γ and PKC, with some possible contribution of the p38 MAP kinase [412]. Basic FGF, which is able to increase the rate of bone formation, stimulates fibronectin expression by binding to FGF-2 receptor and activation of PI-PLCγ2, PKCα, c-Src in rat osteoblasts [413].

#### 5.4.3. Platelet-Derived Growth Factor Induced Signaling Pathway

Platelet-derived growth factor (PDGF) is a potent and selective stimulator of Pi transport in osteoblastic cells. The mechanism responsible for this effect is not mediated by MAP kinases but involves tyrosine phosphorylation-dependent activation of PLCγ and PI-3-kinase [414]. PDGF-mediated chemotaxis of MC3T3-E1 osteoblast-like cells is dependent on both PLC and PI-3-kinase [415]. Moennings *et al.*[416] showed for the first time that PDGFRα signaling stimulates osteogenesis of neural crest cells-derived osteoblasts by activating the PI-PLC-γ pathway. This may suggest an involvement of this pathway in the etiology of human craniosynostosis.

#### 5.4.4. Parathyroid Hormone Induced Signaling Pathway

PTH is known to have both catabolic and anabolic effects on bone. The dual functionality of PTH may stem from its ability to activate two signal transduction mechanisms: adenylate cyclase and PLC [417,418]. In osteoblasts, PI-PLC-β2 transduces the signals from PTH, PGE_2_, and other prostanoids [419,420]. Several reports suggest that PTH activation of PKC, via the stimulation of PLC, plays a role in stimulating the synthesis and release of transforming growth factor-b1 (TGF-b1) [421] as well as in the PTH-stimulated synthesis of insulin-like growth factor binding protein-5 [422]. Both of these factors stimulate bone deposition by activating osteoblast growth and differentiation and may, therefore, play a role in the coupling of bone resorption to bone deposition. Regulation of the PLC pathway through the PTH1R can be significantly increased by elevating the expression of G(11)α in osteoblastic cells. This leads to increased PTH stimulation of MMP-13 expression by activation of AP-1 factors: c-jun and c-fos [423].

#### 5.4.5. PGD_2_ Induced-Signaling Pathway

PGD_2_ stimulates Ca^2+^ influx from the extracellular space and activates phosphoinositide (PI)-hydrolyzing PLC and PC-hydrolyzing PLD independently of PGE2 or PGF_2_α in osteoblast-like MC3T3-E1 cells [251,424]. Thrombin raises Ca^2+^_i_, in UMR 106-H5 rat osteoblast-like osteosarcoma cells by activating PI-PLC [425]. Exogenous PA appears to increase IP_3_ accumulation in osteoblast-like cell line MOB 3-4 by activating PI-PLC [426].

#### 5.4.6. PGE_2_ Induced-Signaling Pathway

U-73122 and calphostin C reduced the PGE_2_-induced phosphorylation of p44/p42 MAP kinase and p38 MAP kinase. These results indicate that PGE_2_ stimulates the induction of HSP27 through PKC-dependent activations of both p44/p42 MAP kinase and p38 MAP kinase in osteoblasts [427]. Bradykinin increased both IL-6 and PGE_2_ synthesis in osteoblastic cells via B2R. PLC, IP_3_-induced Ca^2+^_i_, and MAP kinases were involved in signal transduction in these cells [428]. PGE_2_ evoked a Ca^2+^_i_, rise via a PI-PLC pathway in MC3T3-E1 osteoblasts, particularly in the growing phase [429]. The proton induced COX-2 expression and PGE_2_ production were mediated through the ovarian cancer G protein–coupled receptor/G_q/11_/PLC pathway in human osteoblastic cells [430].

#### 5.4.7. PGF_2_ Induced-Signaling Pathway

PGF_2_α induces phosphoinositide hydrolysis by PLC and PC hydrolysis by PLD through heterotrimeric GTP-binding protein, resulting in the activation of PKC in osteoblast-like MC3T3-E1 cells. PGF_2_α can also stimulate the synthesis of DNA [431,432]. Zinc reduces PGF_2_α-induced IL-6 synthesis via suppression of phosphoinositide-hydrolyzing PLC and PC-hydrolyzing PLD in osteoblasts [433]. It is well known that osteoporosis is a common complication in patients with glucocorticoid excess. Glucocorticoid inhibits PGF_2_α-induced PGE_2_ synthesis through the inhibition of PI hydrolysis by PLC as well as PLA_2_ in osteoblast-like cells [434]. Contrary to sphingosine 1-phosphate (S1P), sphingosine inhibits PGF_2_α-induced phosphoinositide hydrolysis by PLC via p38 MAP kinase in osteoblasts. [435,436].

#### 5.4.8. Vitamin D-Induced Signaling Pathway

Only PI-PLC-β1 linked to a PTX-insensitive G-protein and PLC-β2 coupled to a PTX-sensitive G protein are involved in the effects of calcitriol and 17β estradiol (the hormonally active form of vitamin D,) respectively, on the mobilization of Ca^2+^ from Ca^2+^_i_ stores. [437]. PLC-β1 is the target effector of Gα(q/11), whereas PI-PLC-β2 is only activated by βγ subunits; this specificity may help to generate membrane receptor-specific responses *in vivo*[438]. When osteoblasts are cultured on surfaces of increasing micro roughness, they exhibit decreases in proliferation, increases in differentiation and local factor production, and enhanced response to 1α,25(OH)_2_D_3_. The cells interact with surfaces through integrins, which signal by the same pathways used by 1α,25(OH)_2_D_3_, *i.e.*, they activate PKC via PLC and protein kinase A via PLA_2_. This provides opportunities for crosstalk that may contribute to the synergistic effects of surface roughness and the vitamin D metabolite [439].

#### 5.4.9. Interleukin-1-Induced Signaling Pathway

PI-PLC-β1 is specifically localized in the nucleus of Saos-2 osteoblast, where it is activated when cells are stimulated with IL-1 [440,441]. Saos-2 cells are characterized by the expression of high affinity receptors for IL-lα, which is one of the most potent stimulators of bone resorption [442]. A recent report [443] demonstrated that nuclear activation of PI-PLCβ1 was dependent on its phosphorylation by the MAP kinase. The MAP kinase pathway is implicated in the pathogenesis of RA because it is activated by proinflammatory cytokines, such as TNF-α and IL-1β [444].

#### 5.4.10. Miscelanous Ligand Binding Stimulated PI-PLC in Osteoblasts

*Pasteurella multocida* toxin, a mitogenic toxin, acts to inhibit differentiation, in particular of bone cells. *In vitro*, it prevents the formation of mineralized bone nodules. *P. multocida* toxin action stimulates PLC leading to activation of protein kinase C, an increase in inositol phosphates, and a rise in Ca^2+^_i_[445]. In human osteosarcoma MG63 cells, thymol causes a Ca^2+^_i_ rise by inducing PLC-dependent Ca^2+^release from the endoplasmic reticulum and Ca^2+^ entry via protein kinase C-sensitive store-operated Ca^2+^channels [446]. CGRP a peptide produced locally in bone, and that may act as a cytokine on bone cells, is not coupled to adenylate cyclase but increases Ca^2+^_i_ levels in CGRP receptor-positive OHS-4 osteosarcoma cells, suggesting that in these cells CGRP induces downstream events driven by PLC in these cells [446]. *N*-formyl-methionyl-leucyl-phenylalanine -stimulated osteogenic differentiation of human mesenchymal stem cell which was mediated via the *N*-formyl peptide receptor-PLC/PLD-Ca^2+^-calmodulin-dependent kinase II-ERK-CREB signaling pathways [447]. The pretreatment of human osteoblast SaM-1 cells with U-73122, a PLC inhibitor, stopped IL-6 and IL-8 synthesis in response to extracellular LPA. The proposed mechanism may involve activation of PLC and IP_3_-mediated Ca^2+^_i_ release in human SaM-1 cells [448].

#### 5.4.11. Purinergic and Serotonin-2 B Receptors

Under certain stress conditions that lead to release of nucleotides from the rat osteoblastic cell line ROS-A 17/2.8, the stimulation of specific purinergic receptors such as P2Y_2_, sensitizes mechanical stress activated Ca^2+^ channel through a mechanism that involves PI-PLC activation. [449]. U-73122 and thapsigargin, a calcium-pump inhibitor, both significantly inhibited the increase in Ca^2+^_i_ induced by extracellular ATP in TBR31-2 cells. This suggests that the increase in Ca^2+^_i_ is due to Ca^2+^_i_ release from the calcium store following activation of PLC [450]. Mice knocked out for the serotonin-2B receptor (5-HT_2B_R) show defects in bone homeostasis. In C1 osteogenic cells the positive action of 5-HT_2B_R on TNAP downstream from eicosanoids requires the activity of the glycosyl-PI-solubilizing enzyme PI-PLC [250].

### 5.5. Presence of PI-PLC in Osteoclasts

During bone resorption osteoclasts remove large amounts of ECM. At the beginning of the resorption cycle, the plasma membrane in contact with the bone expands through fusion of lysosomes and intracellular vesicles into the convoluted ruffled borders. Consistent with the function of osteoclasts, some of the PI-PLC identified in osteoclasts are involved in membrane trafficking and in cytoskeletal rearrangement. PI-PLCγ2 modulates bone homeostasis by affecting osteoclast recruitment and function. PI-PLCγ2 is implicated in actin cytoskeletal reorganisation in osteoclasts and neutrophils. It is an important regulator of α(v)β(3) integrin-mediated bone osteoclast cell adhesion, migration, and in bone resorption [346,451,452]. Although PI-PLCγ1 is expressed in osteoclasts, it cannot compensate for the absence of PI-PLCγ2 [346], suggesting that PI-PLCγ1 and PI-PLCγ2 are implicated in distinct signaling pathways. Experimental evidence of the presence of PI-PLC in osteoclasts arises from the analysis of the mechanisms induced by endocrine and paracrine factors that regulate osteoclast formation and activity. Such factors include calcitonin, RANK ligand, Ca^2+^, H^+^, nucleotides regulating osteoclast activity in several ways. Very often, the PLC activity in osteoclasts was evidenced by the use of PLC inhibitors such as U-73122.

#### 5.5.1. Calcitonin Induced Signaling Pathway

Calcitonin inhibits the activity and changes the morphology of osteoclasts by interfering with trafficking to and from the ruffled border [453,454]. Such action makes it a possible therapeutic target for the treatment of osteoporosis [455]. Using U-73122, PLC was shown to be implicated in the inhibition of endocytosis from the ruffled borders of rabbit osteoclasts induced by a calcitonin treatment. The PLC inhibitor reversed the calcitonin effect and restored endocytic trafficking to the level equivalent to 75% of that in untreated controls [456].

#### 5.5.2. Intracellular Ca^2+^ Induced Signaling Pathway

**Figure 7:**
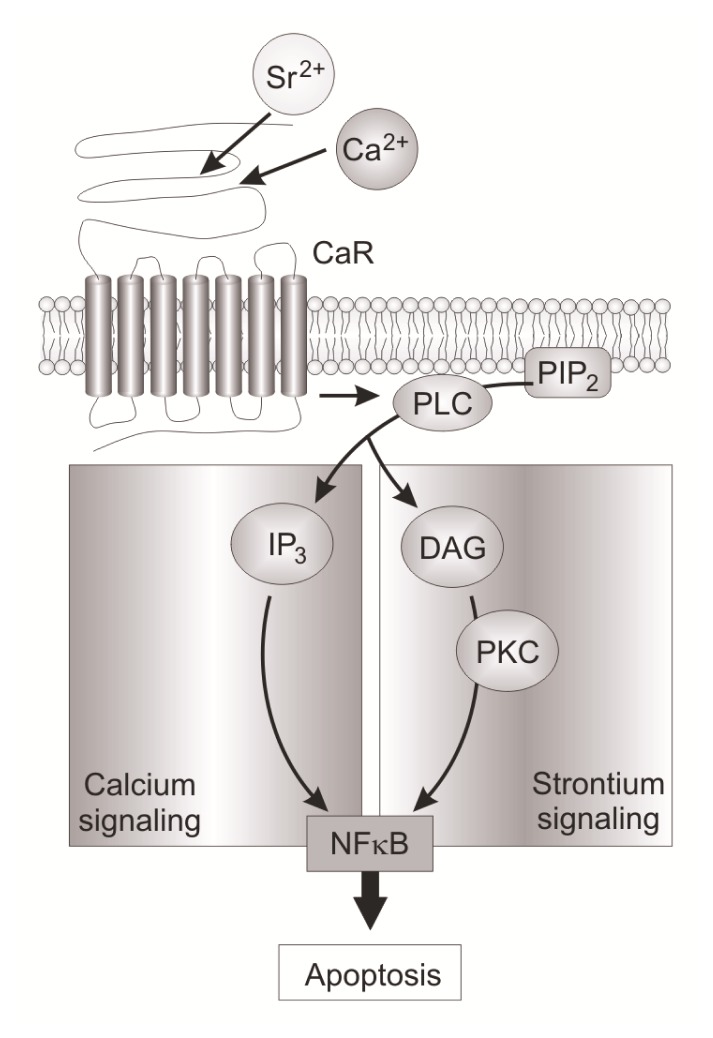
Calcium and strontium induce distinct intracelular calcium signaling. Upon stimulation by extracellular calcium, calcium-sensing receptor (CaR) activates PLC, which is responsible for the translocation of nuclear factor κB (NF-κB) from the cytoplasm to the nucleus in mature osteoclasts, in an IP3-dependent manner. Upon stimulation by extracellular strontium, CaR also activates PLC inducing the DAG-PKCβII signaling pathway and then promoting translocation of NF-κB from the cytoplasm to the nucleus in an IP3-independent manner. Taken from [465].

It is essential to make a distinction between the effect of Ca^2+^_e_ in osteoclasts that may stimulate the rise in Ca^2+^_i_ in osteoclasts and the effect of other ligands that may induce an increase in Ca^2+^_i_ concentration. The latter may affect osteoclast differentiation and function differently although both may involve PLC activity. Increasing Ca^2+^_e_ to levels comparable to those resulting from local bone resorption inhibits osteoclast differentiation and osteoclastic bone resorption [457]. Osteoclasts can sense increasing levels of Ca^2+^_e_, which in turn trigger a rapid rise in the cytosolic calcium concentration, disassembly of podosomes, and osteoclast apoptosis [458–461]. RANKL induced an increase in Ca^2+^_i_ of extracellular origin resulting of the opening of calcium channels—possibly transient receptor potential vanilloid channels 5—on the surface of human osteoclasts [462]. Ca^2+^_i_ oscillation could be triggered by a RANKL-dependent receptor potential vanilloid channels 2 calcium channel in preosteoclast RAW264.7 cells [463]. Similarly to RANKL, Ca^2+^_e_ (20 mM) appeared to trigger rapid and significant nuclear translocation of NF-κB in a CaR- and PLC-dependent manner [464,465] (Figure 7). The CaR is coupled to PLC activity that induces an increase in Ca^2+^_i_ concentration. Sr_e_^2+^—which exerts both an anti-catabolic and an anabolic effect on bone cells—acts through CaR and induces osteoclast apoptosis through a signaling pathway similar to but different in certain respects from that of Ca_e_^2+^ (Figure 7) [465]. The cation sensing by osteoclast-like GCT23 cells is mediated by a PLC-coupled receptor [466]. Osteoclast activity is inhibited by elevated Ca^2+^_e_ that induce a PLC-dependent rise in Ca^2+^_i_ Lyn [467,468] as well as leucine-rich repeat-containing 17 (LRRc17) [469]—by interfering with the PI-PLC-γ1—both down-regulated Ca^2+^_e_ signaling and inhibited osteoclast differentiation [467]. In constrast to the inhibition effects on osteoclasts induced by Lyn or LRRc17, calcitonin increases Ca^2+^_i_ via a PLC-PKC-calcium signaling pathway, stimulating endocytosis in osteoclasts [456]. Several reports confirmed the involvement of PLC in the increase in Ca^2+^_i_. U-73122 blocks the calcium sensitive component in avian osteoclasts [470]. Extracellular nucleotides caused elevation of Ca^2+^_i_ in osteoclasts—isolated from femora and tibiae of rat or rabbit pups—by activating P2Y receptors [471]. Inhibition of PLC with U-73122 or inhibition of endoplasmic reticulum Ca^2+^-ATPase with cyclopiazonic acid or thapsigargin abolished the rise of Ca^2+^_i_ induced by the binding of nucleotides to P2Y receptors [471]. RANKL acting on osteoclasts isolated from the long bones of neonatal Wistar rats or or New Zealand white rabbits elevated Ca^2+^_i_ in Ca^2+^-containing and Ca^2+^-free media. The increase in Ca^2+^ was prevented by U-73122 [472,473]. It was suggested that PI-PLC-γ could regulate Ca^2+^ channel during RANKL-signaling for terminal differentiation of osteoclasts and that RGS12 was essential for the terminal differentiation of osteoclasts induced by RANKL [474]. It was proposed that PI-PLC-γ2 mediates RANKL-induced osteoclastogenesis and is a potential candidate for the antiresorptive therapy [475]. Other proteins such as RGS10 can mediate PLC activation and Ca^2+^_i_ oscillations [474]. The Ca^2+^_i_ calmodulin complex competes for the PIP_3_-binding site on RGS10 and frees the bound PIP_3_. Once the Ca^2+^_i_ concentration reaches its peak, Ca^2+^_i_ begins to reload into the endoplasmic reticulum and the Ca^2+^_i_ calmodulin complex dissociates from RGS10 at the low Ca^2+^_i_ concentration. Free PIP_3_ activates PLC and then binds RGS10 again. PLC activation triggers a release of Ca^2+^_i_ from intracellular stores by generating IP_3_ to induce a second peak. This process continues to repeat itself, causing Ca^2+^_i_ oscillations. RGS10 mediates PLC activation and Ca^2+^_i_ oscillations through its Ca^2+^_i_ -dependent dual interaction with Ca^2+^_i_ calmodulin and PIP_3_[474].

#### 5.5.3. Osteoprotegrin Induced Signaling Pathway

OPG-inhibitor of osteoclast differentiation—directly binds to RANKL, whereas LRRc17 acts as a negative regulator of RANKL-induced murine osteoclast differentiation by blocking PI-PLCγ signaling [469]. PLC exerts indirect effect in endothelial cells of the bone vasculature, modulating development, remodeling, and bone repair by secreting OPG which acts on osteoblastic and osteoclastic lineage cells. For example, IL-4 and IL-13 induced OPG expression through activation of a PLC-sensitive STAT6 pathway in human umbilical vein endothelial cells [476].

#### 5.5.4. RANK Induced Signaling Pathways

**Figure 8:**
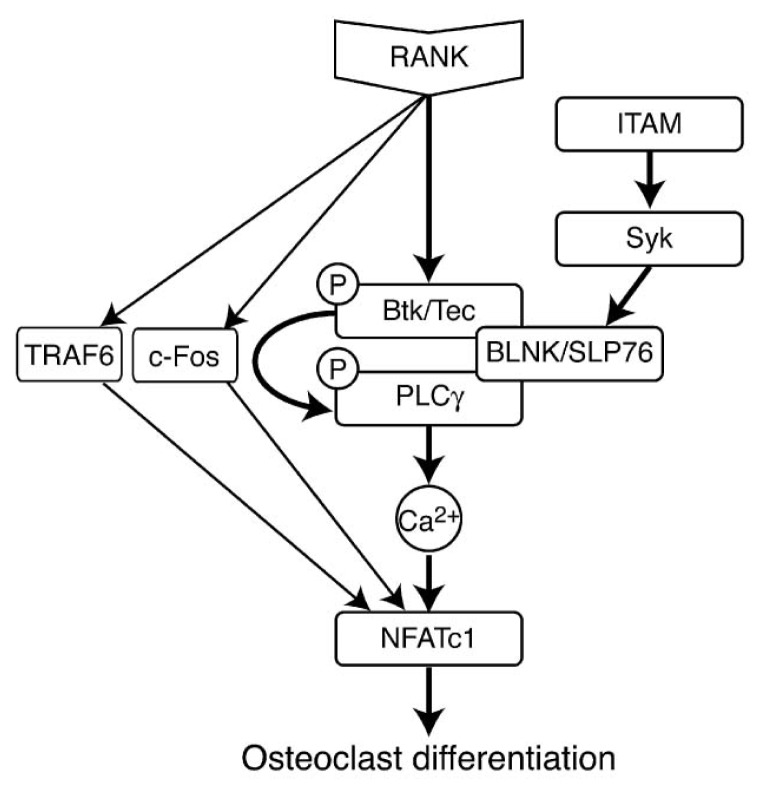
Integration of the RANK and ITAM Signals by Tec Kinases. RANKL binding to RANK results in activation of pathways involving TRAF6 and c-Fos. Tec kinases are phosphorylated by RANK. ITAM phosphorylation results in the recruitment of Syk, leading to activation of adaptor proteins such as BLNK and SLP-76, which function as scaffolds that recruit both Tec kinases and PLCγ to form the osteoclastogenic signaling complex. This complex induces calcium signaling required for the induction and activation of NFATc1, the transcription factor for osteoclast differentiation. Adapted from [477].

Mice lacking the tyrosine kinases Btk and Tec show severe osteopetrosis caused by a defect in bone resorption indicating that Btk and Tec kinases are crucial in the regulation of osteoclast differentiation [477]. RANKL induced tyrosine phosphorylation of PI-PLCγ1 and PI-PLCγ2 was markedly suppressed in *Tec*^−/−^*Btk*^−/−^ cells. Taken together the findings suggest that RANKL binding to RANK results in activation of classical pathways involving TRAF6 and c-Fos. In addition, Tec kinases are phosphorylated by RANK (Figure 8). ITAM phosphorylation results in the recruitment of Syk, leading to activation of adaptor proteins such as BLNK and SLP-76, which function as scaffolds that recruit both Tec kinases and PI-PLCγ to form the osteoclastogenic signaling complex (Figure 8). This complex is required for calcium signaling to activate NFAT1, the key transcription factor for osteoclast differentiation [477]. A molecular mechanism for the long-term link between RANK and ITAM signals has been proposed. A domain in RANK is dispensable for the early phase of RANK and ITAM signaling but is essential for the late-phase signaling, which involves PI-PLC-γ2 binding to RANK [478] (Figure 8). A linker for activation of T cells (LAT), regulates RANKL-induced osteoclast differentiation and is involved in RANKL-induced PI-PLC-γ activation and NFAT1 induction [479]. PI-PLCγ1 is also involved in RANKL-induced Ca^2+^ oscillations as shown by marked inhibition of the oscillations in BM-derived monocyte/macrophage precursor cells in which PI-PLCγ1 was knocked down with PLCγ1 siRNA [480]. The absence of PI-PLC-γ1 is not compensated by the presence of PI-PLCγ2, suggesting that both PI-PLC-γ1 and PI-PLC-γ2 participate in RANKL-induced Ca^2+^ oscillations. RANKL induced a significant increase in Ca^2+^_i_ of extracellular origin, probably as a result of the opening of TRPV-5 calcium channels on the surface of human osteoclasts. Mutant forms of SH3BP2—occuring in patients with cherubism- potentiate RANKL-induced phosphorylation of PI-PLC-γ isoforms, suggesting that SH3BP2, as well as PLC-γ2, are potential targets in the treatment of disorders characterized by excessive osteoclastic development [481,482].

#### 5.5.5. Parathyroid Hormone Induced Signaling Pathway

As in most cells expressing the PTH/PTH-related peptide receptor in cells, stimulation with with PTH agonists results in the activation of two G protein-dependent signaling pathways, the Gα_s_/adenylyl cyclase/cAMP/protein kinase A pathway and the Gα_q/11_/PLC/IP_3_/Ca^2+^/PKC pathway, as many other GPCRs, activates several signaling pathways, including the G_q_/_11_-linked PI-PLC-PKC signaling pathway as determined in cell cultures. However, there are only few reports investigating whether such signaling occurs *in vivo*[483]. Estrogens modulate the catabolic effects of PTH on bone *in vivo* and *in vitro*. Estrogens suppress PTH-stimulated osteoclast-like cell formation by blocking both the cAMP-dependent protein kinase A pathway and the PLC-coupled calcium/PKC pathway [304,484].

### 5.6. Presence of PI-PLC in Smooth Muscle Cells and Possible Roles

Most of experimental evidence on the presence of PI-PLC in smooth muscle cells originates from analysis of signaling pathways [485–487] and the use of general PLC inhibitor U73122 or PLC isotype inhibitors D609 and ET-18-OCH3. No evidence was obtained by Western blotting for the presence of PLC-β, PLC-γ and PLC-δ in bovine mesenteric lymphatic smooth muscle cells. However, a PLC activity was concentration-dependently stimulated by Ca^2+^[488]. In rat thoracic aortic smooth muscle cell, vasopressin induces V1 receptors to release AA, DAG and PChol via activation of both a PI- and PC-PLC [489]. Both isotypes of PLC were involved during VSMC proliferation [490]. S-1-P in Rat arterial VSMC induced time-dependent activation of PI-PLC-β as evidenced by the use of U-73122 [491].

### 5.7. Presence of PI-PLC in Odontoblasts and Possible Roles

Odontoblasts—extracted from dental pulp of newborn Wistar rats—demonstrated an IP_3_-induced Ca^2+^ release activated by PLC-coupled receptors [492]. Calcium ions and PLC were required for the capsaicin-induced expression of OPG in human periodontal ligament, which is known to play an important role in the bone-remodeling process [493]. U73122 was able to ablate the basic FGF-induced neuronal differentiation of dental pulp stem cell (DPSC) and the authors suggested that basic FGF-induced neuronal differentiation of DSPC could involve a PI-PLC-γ pathway [494].

### 5.8. Genetic Models

Genetically manipulated mice revealed that the lack of some PLC isozymes causes defects in fertilization and development of the circulatory, hematopoietic, immune, and skin systems [495]. PI-PLC-ζ and PI-PLC-δ4 are critical in fertilization [495]. Liao *et al.*[496] reported that vasculogenesis is impaired in PLC-1 knockout embryos. PI-PLC-δ1/-δ3 knockout mice show mainly placental vascular defects [497]. PI-PLC-β3 plays some role in angiogenesis [498]. PI-PLC-γ1 is essential for renal development and for the development of hematopoietic stem cells [499]. PLC-γ1 and PLC-γ2 play important roles in the immune system, in development of B cells [500] as well in in bone homeostasis [501–506]. PI-PLC-γ2 null mice (*PLC-γ2*^−/−^) are osteopetrotic, *i.e.*, shox features of a hereditary disease marked by abnormally dense bone, and by the common occurrence of fractures of the affected bone. [452]. PI-PLC-γ2 knockout mice have less osteoclasts due to defective upregulation of NFAT2, which is a critical transcription factor activated by RANKL that controls osteoclast differentiation [507]. These findings indicate that PLC-γ2 regulates osteoclastogenesis as a downstream effector of RANKL in mice. PI-PLC-γ2 is essential for RANK signaling, and its deficiency leads to defective lymph node organogenesis and osteoclast differentiation [508]. PI-PLCγ2 activation/function may provide opportunities to develop targeted therapeutic approaches for treatment of inflammatory and osteolytic diseases. However, *PLC-γ2*^−/−^ mice proved that PI-PLC-γ2 is not a major player in ovariectomy-induced bone loss, indicating that PLC-γ2 may not be a suitable therapeutic target in postmenopausal osteoporosis [509].

## 6. PLC-Related but Catalytically Inactive Protein

PLC related but catalytically inactive protein (PRIP) is a novel molecule in bone biology research and was originally identified as IP_3_-binding protein. This protein is similar to PLC-δ1 but is catalytically inactive [510,511]. The PRIP family consists of at least two types of proteins (PRIP-1 and PRIP-2 subfamilies). PRIP has a number of binding partners, including the catalytic subunit of protein phosphatase 1α (PP1α) and PP2A [512,513] and the phosphorylated (active) form of Akt [514] in addition to IP_3_ and PIP_2_[515]. PRIP gene-deficient mice (*prip*^−/−^), genetically deficient in type 1 or type 2 isoforms or both, brought light the physiological functions of PRIP: modulation of GABAA receptor signaling [516], dysfunction of reproduction, negative regulation of multiple-hormone secretion [517] and bone properties [518]. PRIP is involved in the phosphorylation-dependent modulation of exocytosis in PC-12 cells [519]. Exocytosis of various peptide hormones, such as gonadotropins and insulin, was up-regulated in *prip*^−/−^ mice, indicating that PRIP is likely to be involved in dense core vesicle exocytosis in a negative manner. PRIP is implicated in the regulation of bone formation in a negative manner, partly through the regulation of SMAD phosphorylation [518]. Since PC-PLC contributes to the progression of atherosclerosis, it was proposed that pharmacological blockade of PC-PLC is a possible approach to atherosclerosis therapy.

## 7. Sphingomyelinase

### 7.1. Groups, Subgroups and Specificity

Sphingomyelinase (SMase), which may be considered as a subtype of PLC, cleaves SM (ceramide phosphorylcholine) to yield ceramide and PChol (Figure 3D). Ceramide, subsequently metabolized by ceramidase and sphingosine kinase to sphingosine and S1P, respectively, appeared to be a lipid second messenger in programmed cell death, cell differentiation and cell proliferation [368,520,521]. There are at least five isoforms of acidic, neutral, and basic SMases differing mainly on pH profiles, cation requirements, and cellular localization. *Smpd3* encodes neutral SMase 2, a membrane-bound enzyme, and is highly expressed in bone. A local neutral SMase2 (also called sphingomyeline phosphodiesterase 3 (SMPD3)) activity is required for a normal bone mineralization and for physiological apoptosis of hypertrophic chondrocytes in the cartilage during early skeletal development [522].

### 7.2. Presence of Sphingomyelinase in Chondrocytes and Possible Roles

Six years ago it has been shown for the first time [523], that articular chondrocytes express both acidic and neutral SMases and are able, in response to the appropriate external signal, to raise levels of endogenous ceramide; depending on which SMase is activated, an inflammatory (neutral SMase) or apoptotic (acidic SMase) response is observed. SMase is implicated in both chondrocyte apoptosis and ECM degradation during cartilage degeneration [524]. Ceramide stimulated synthesis of specific MMPs which in turn induced degradation of ECM and cell death in cartilage. This suggests that the SMase pathway could participate in vascular invasion of the growth plate by disrupting cartilage-ECM homeostasis, resulting in down-regulation of the type II collagen [524]. In addition, some ceramide metabolites have been implicated in the cartilage degradation and arthritic disease. For example, in Farber’s disease, the lack of ceramidase causes excessive accumulation of ceramide within the cartilage and bone, and is associated with joint pain and arthritis-like joint degeneration [525]. SMase down-regulates type II collagen in articular chondrocytes via activation of the ERK signaling cascade, redistribution of SOX9, and recruitment of c-Fos [526]. These findings provide direct evidence for a role of SMase metabolites in human arthritic disease. This enzyme could represent a target for pharmacological intervention against cartilage loss in arthritic diseases.

### 7.3. Presence of Sphingomyelinase in Osteoblasts and Possible Roles

Information regarding the effects of ceramide in cells of skeletal origin is limited and conflicting. Ceramide *in vitro* may be either a pro-death agent or it may protect cells, depending on the experimental conditions. Moreover, TNF-α, IL-1, platelet-derived growth factor and vitamin D3 as potent regulators of bone remodelling may utilize sphingosine metabolites such as ceramide or S1P as second messengers in their respective signal transduction pathways via activation of SMase. This suggests that sphingosine metabolites are operating as intracellular signaling molecules in osteoblasts and osteoclasts. In fact, these metabolites are able to mimic the biological actions of the above cytokines in osteoblasts. The proapoptotic agent TNF-α has been reported to induce osteoblast cell death in a process involving ceramide [527]. However, conversely, ceramide was shown to be mitogenic in MC3T3-E1 osteoblast cells [528]. Among SM metabolites, ceramide enhances the BMP stimulated osteocalcin synthesis in osteoblasts and its effect is exerted at a point upstream from p44/p42 MAP kinase [529]. Scyphostatin—a neutral SMase inhibitor—revealed that neutral SMase-induced release of ceramide directly activated the intrinsic mitochondrial apoptotic pathway [530]. Ceramide signals osteoblast survival and apoptosis through different intracellular pathways, and alteration in the intracellular levels of ceramide may play an important role in bone remodeling [531]. S1P acts as a second messenger for tumor necrosis factor-α-induced synthesis of IL-6 in MC3T3-E1 cells and the p44/p42 MAP kinase is involved in the signaling [330,435,532,533]. It has been shown that not ceramide but sphingosine and S1P transiently mobilize Ca^2+^_i_ from intracellular stores in osteoblast-like MC3T3-E1 cells [534].

### 7.4. Presence of Sphyngomyelinase in Osteoclasts and Possible Roles

Sphingolipid metabolism is implicated in osteoclastogenesis. Acid SMase gene was identified as a gene induced by NFAT2 during the late stages of osteoclastogenesis [535]. SMase and C2 ceramide inhibited bone resorption by suppressing osteoclast activity through suppression of F-actin ring formation essential for ruffled border formation [536].

### 7.5. Genetic Models

Bone deformities in mouse models lacking a functional *Smpd3* gene underscore the importance of sphingolipid metabolism in skeletal tissues [522,537,538]. Stoffel *et al.*[538,539] characterized the skeletal phenotypes of the *Smpd3*^−/−^ mice as chondrodysplasia and speculated a systemic role for neuronal SMPD3 in the regulation of the skeletal development.

### 7.6. Effects of Sphyngomyelinase Metabolites at Matrix Vesicle Level

SM is a structural component of MVs released by pre-hypertrophic/upper hypertrophic chondrocytes. SM is specifically enriched in MVs as compared to plasma membrane of growth plate chondrocytes from which they derive, Table 1[53]. During cartilage MV-induced mineralization, there is a progressive disappearance of SM [53]. It was suggested that a neutral SMase-2 could be a possible candidate for the SM hydrolysis [53]. Indeed, SMPD3 has been identified in MVs isolated from osteoblast-like Saos-2 cells [540]. The hydrolytic activity of SMPD3 may serve as additional source of P_i_, since SMase produces PChol which is hydrolysed by PHOSPHO1 present in MVs [47,523,541]. This gives rise to the possibility of a novel mechanism by which phosphate may be unleashed through the action of PHOSPHO1 and SMase such as SMPD3 to contribute to the changes in P_i_ concentration inside MV lumen. Exosome formation in multivesicular bodies is triggered by hydrolysis of sphingolipids and release of ceramide. This reaction is catalyzed by SMPD3 and an inhibitor of SMPD3, GW4869, efficiently abrogates exosome release in the oligodendroglial cell line OLI-neu.

## 8. Phospholipase D

### 8.1. Groups, Subgroups and Specificity

PLD belongs to a large superfamily of enzymes which hydrolyzes the phosphodiester bonds of membrane phospholipids, producing PA and polar head group (Figures 2,3B). A large subset of enzymes with PLD activity share a conserved HxKx_4_Dx_6_GSxN motif (HKD) [542] or a variation of thereof, which is responsible for catalytic activity [77]. Non-HKD enzymes—such as glycosyl-PI specific PLD (GPI-PLD), *N*-acyl PE-PLD, cytochrome P450 1A2 and 2E1 as well as ATX- have a PLD activity but have divergent structures and catalytic mechanisms [77] (Table 8). PA is also produced by a DAG kinase from DAG or by LPA acyltransferase from LPA. Alternatively, PA can be also be synthesized by sequential enzyme catalyzed alcylation from glycerol-2-phosphate [77]. PA—due to its small negatively charged group—binds to protein and facilitates changes in lipid bilayer and therefore is implicated in vesicular trafficking, exocytosis and endocytosis [543,544]. PA is also precursor to other lipid signaling molecules such as DAG and LPA. DAG is a well known activator of PKC [545–548]. To date 10 isotypes of PKC have been identified, which are subgrouped in three categories: classical PKC (PKC-α, -β and -γ) require Ca^2+^, DAG and phospholipids; novel PKC (PKC-δ, -ɛ, -η and -θ) are Ca^2+^-independent but DAG and phospholipid dependent; and atypical PKC (PKC-ζ, -1/λ and -μ) are insensitive to Ca^2+^ and DAG [549]. DAG can be converted to AA, a precursor of eicosanoids [550]. In addition, PA—as a lipid messager, can interact with several signaling proteins including Raf-1 [551,552] and the mammalian target of rapamycin (m-TOR) [553]. PA is involved in signaling cascades affecting cell- growth, proliferation and survival [77]. PLD catalyzes the reaction of transphosphatidylation using water or primary alcohols (ethanol or 1-butanol) as nucleophiles to generate PA, phosphatidylethanol or phosphatidylbutanol, respectively [554]. PLD activity has been evidenced in various organisms, including plants, mammals, bacteria and yeast. In humans, two genes, *pld1* and *pld2*, encoding the PLD enzyme were found. *pld1* encodes the 124 kDa protein PLD1a (1074 amino acids), and an alternatively spliced form PLD1b (1036 amino acids), which lacks 38 amino acid residues, the most studied variants. There are two other PLD1 splice variants PLD1c and PLD1d. *PLD2* encodes a 106 kDa protein with 50% homology to PLD1 [77,554]. PLD2 has three variants PLD2a, 2b and 2c (Table 8). The PLD2b variant lacks 11 amino acids in its C-terminus compared to PLD2a, but it is still functional [555]. Both isoforms are capable of hydrolyzing PC, PE, PS but are not capable of hydrolyzing PI, PG or cardiolipin [77]. In addition, PLD can hydrolyze LPC and LPS and produce LPA. Two other mammalian PLD enzymes have been identified with significant sequence homology to viral PLD: PLD3 or Hu-K4 [556] and an endonuclease-like mitochondrial PLD enzyme [557]. PLD3 activity has not been detected [77], while mitochondrial PLD hydrolyzes cardiolipin to generate PA [557]. It is usually stated that PLD1 and PLD2 are expressed in nearly all mammalian tissues [77,558] and that PLD plays an important role in modulating cellular function [559]. However, very little is known about the presence and function of PLD in osseous tissues. Although both isoforms catalyze the same reactions and utilize similar substrates to generate PA or transphophatidylation species, they have usually distinct subcellular localizations [77]. As reviewed [77], it is generally accepted that PLD1 is localized to perinuclear membranes, including early endosomes and Golgi, under basal conditions [560]. Upon stimulation, PLD1 translocates to the plasma membranes or late endosomes [77]. PLD2 is usually located in the plasma membrane under basal conditions and translocates to the recycling vesicles [77]. PLD2 also binds to β-actin [561]. PLD1 [562] and PLD2 [563] are palmitoylated at two cystein residues and both contain PH and phox homology lipid binding domains. The palmitoylation and the two lipid binding domains contribute to the association of PLD with membrane lipids [554]. PLD activity is regulated by many factors, including phosphoinositides. PLD1 has a low basal activity and is extensively regulated by PKC and members of the ARF and Rho (RhoA, Rac1, Cdc42) families of small GTPases. PLD2 has a higher basal activity than PLD1 but has been shown to respond to ARF and PKC [564]. It has been reported that PLD/PA can directly activate regulatory proteins playing key roles in cell physiology, such as PI-4-phosphate 5-kinase, PKC, PLCγ, Raf-1 kinase and MAP kinase [565]. These proteins are also considered as candidates mediating cellular signaling during osteoblast proliferation and differentiation [566] but also during osteoclast differentiation [567]. PLD and its enzymatic product, PA, regulate the actin cytoskeleton, vesicle trafficking for secretion and endocytosis, and receptor signaling [568]. Free choline is not thought to fulfil any intracellular signaling roles [568]. Although PLD is important for many physiological processes, its function in bone metabolism is unclear. Their presences in chondrocytes and in osteoblasts have been reported.

### 8.2. Presence of PLD in Chondrocytes and Possible Roles

A PKC-regulated PLD activity stimulated by phorbol 12-myristate 13-acetate (a known PLD stimulator) has been evidenced in chondrocytes. This activity could be inhibited with staurosporine—a PKC inhibitor [569]. Other experimental evidence of PLD presence in chondrocytes, which provided more insight into the possible roles of PLD in biomineralization, originated from the determination of growth plate chondrocyte regulation by vitamin D3 metabolites [570]. 1α,25-(OH)_2_D_3_ and 24R,25-(OH)_2_D_3_ vitamine D3 metabolites are found in growth plate cartilage, indicating that they are implicated in regulation mechanisms of growth plate cartilage. Indeed, in the absence of vitamin D3, the growth plate fails to mineralize and the hypertrophic zone becomes enlarged [571,572]. Not only chondrocytes but osteoblasts produce 1α,25-(OH)_2_D_3_ and 24*R*,25-(OH)_2_D_3_ which may function as autocrine regulators of matrix events, including MV formation, enzyme activity and matrix protein remodelling during longitudinal growth, calcification, and growth factor activation [573]. The growth plate is an ideal model since the lack of a vasculature ensures that only one cell type, the chondrocyte, is present in the growth plate. The cells can be subdivided into maturation zones (post-proliferative, pre-hypertrophic and upper hypertrophic zones so-called “growth zone”) and resting zones [387]. Using rat costochondral growth zone and resting zone chondrocytes cultures, it has been shown that resting zone chondrocytes respond preferentially to 24*R*,25-(OH)_2_D_3_[574,575], while hypertrophic chondrocytes respond preferentially to 1α,25-(OH)_2_D_3_[317,576]. Vitamin D3 metabolite, 1α, 25-(OH_2_)D_3_, caused stimulation of PKC activity via a PI-PLC in growth zone chondrocytes [390,391]. In resting cells, 24*R*,25-(OH)_2_D_3_ caused also a rapid increase of PKC activity but the mechanism involved was independent of PI-PLC [577]. 24*R*,25-(OH)_2_D_3_ exerts its effect through a vitamin D receptor [578] resulting in activation of PLD2 (based on G-protein-independent property) [391,570] and production of LPA [262]. Both pathways produce DAG and cause PKC activation but their time course differs [579]. The mechanisms that render the 1α,25-(OH_2_)D_3_ pathway silent in resting zone chondrocytes and the 24*R*,25-(OH)_2_D_3_ pathway silent in growth zone chondrocytes is controlled by a PLA_2_ activity. Inhibition of PLA_2_ blocks the effect of 1α,25-(OH_2_)D_3_ on growth zone cells while activation of PLA_2_ with melitin mimics the effects of 1α, 25-(OH_2_)D_3_ on growth zone cells [580]. Inhibition of PLA_2_ activates PKC and mimics the effect of 24*R*,25-(OH)_2_D_3_ on resting zone cells whereas activation of PLA_2_ blocks the effect of 24*R*,25-(OH)_2_D_3_ on PKC [581]. RT-PCR and Northern blot analysis revealed the presence of PLD1a, PLD1b and PLD2 mRNAs in both resting zone and growth zone chondrocytes. PLD activity was detected in both resting zone and growth zone chondrocytes and could be inhibited by wortmannnin—a known PLD inhibitor [570]. PLD activity stimulated by 24*R*,25-(OH)_2_D_3_ in resting chondrocytes may have two functional roles. The first one is an indirect increase of DAG (which is not obtained via PLC) which activates PKC, increases MV production [582], maturation and cell survival [583]. So far it is not clear how DAG is produced in this pathway. The second hypothetical role of the PLD stimulation by 24*R*,25-(OH)_2_D_3_ in resting chondrocytes could be evoked is an increase of LPA that could then bind in an autocrine manner to the LPA1 or LPA3 receptor [262,397]. Although there is no dispute that resting zone chondrocytes contain intracellular and secrete extracellular LPA (among them, 1-oleoyl-2-hydroxy-*sn*-glycero-3-phosphate), which can be activated through a membrane-associated vitamin D receptor [578], the possible pathway of LPA production from PA needs to be ascertained. So far, there is no experimental evidence that the production of PA catalyzed by PLD in the resting chondrocytes is the only source of secreted LPA. Indeed no information on the type of PLA_2_ implicated in the hydrolysis of PA forming LPA in resting zone chondrocytes is reported for this pathway. Alternate pathways for the 24*R*,25-(OH)_2_D_3_ induced LPA production need to be considered. The actin cytoskeleton plays an essential role in adhesion and PLD is physically and functionally linked to actin cytoskeleton [584]. It has been reported that the release of MVs from cultured epiphyseal chondrocytes was correlated with changes in cellular actin distribution [309]. PA—the product of hydrolysis of phospholipids by PLD is a fusogenic lipid [585], implicated in different steps of vesicular trafficking and intracellular membrane fusion events [586–588]. Laulagnier *et al.*, 2004 [589] have observed the enrichment of active PLD2 on exosomes secreted by RBL-2H3 cells and shown that PLD2 was necessary to obtain maximal exosome secretion. Taken together the overall findings may suggest that PLD-dependent remodelling of actin cytoskeleton could participate in promoting MV formation from chondrocytes as well as from osteoblasts.

### 8.3. Presence of PLD in Osteoblasts and Possible Roles

The earliest experimental evidence of PLD activity in osteoblasts and its regulation originated from osteoblast-like MC3T-E1 cells. Despite that the fact that a lot of information on the activation of PLD is available, little is known about the possible role of PLD in osteoblasts. In these cells, PLD can be activated by PKC or in a Ca^2+^ dependent manner. PLD is activated in a PKC dependent manner by the platelet-derived growth factor [590] and by thromboxane A2 [591], while PLD is activated Ca^2+^ dependently by PGD_2_[424,592], PGE_2_[593], extracellular ATP [559] and thrombin [594]. PLD is activated Ca^2+^ dependently by PGF_2_[595] and independently of the activation of PKC [596], while retinoic acid suppresses the PLD activity activated by PGF_2_[597]. Tyrosine kinase may regulate PLD activity in these cells [598,599]. Other factors such as ET-1 [600,601] and basic FGF [602] activate PLD activity in osteoblast-like MC3T-E1 cells independently of PKC. NaF activated PLD and induced Arf/Rhoa translocation in osteoblast-like Saos-2 cells [603]. More information on possible functional roles of PLD is gained from osteoblast-like UMR-106 cells. A phorbol 12-myristate 13-acetate treatment of osteoblast-like UMR-106 cells activated PLD and lead to the production of PGE_2_ but not PGF_2_α [604] confirming for the first time that in osteoblasts, PA can be converted in PGE_2_ via a PLD/phosphatidate phosphohydrolase/DAG lipase/COX pathway [605–607]. Arachidonate metabolites such as PGE_2_ were found to play an important role in bone and cartilage metabolism [608]. These findings reveal a new aspect of PLD action [604] as a possible mediator in bone metabolism. Other factors also revealed the functional roles of PLD in osteoblasts. For example, PTH stimulates bone formation by preventing osteoblast apoptosis [609] and by activating diverse signaling pathways. PTH can stimulate PLD activity in UMR-106 cells [605–607]. Another example is provided by epidermal growth factor (EGF) which participates in the regulation of bone resorption in mice and mouse calvaria *in vitro* organ cultures [610]. EGF activates PLD signaling cascade in osteoblasts from Sprague-Dawley 21-day fetal rat calvaria, suggesting a general mechanism of PLD signaling pathway in osteoblasts [611]. MG63 osteoblast-like cells showed increased PLD activity, phosphatase alkaline activity and osteocalcin production on sandblasted titanium surface suggesting that PLD regulates osteoblast differentiation [612]. PLD1 activity may promote adhesion-dependent osteoblast differentiation response [612]. It was reported that both PLD1 and PLD2 can mediate the response of osteoblasts to surface microstructure although they did so in a different manner [613]. PLD, by virtue of producing PA, could turn up the mineralization process by affecting P_i_ concentration because human alkaline phosphatase isoenzymes are able to hydrolyze phosphatidates with various fatty acyl chains (e.g., phosphatidate and dioleoyl, distearoyl, dipalmitoyl, dimyristoyl and dilauroyl phosphatidates) [614]. On the other hand it has been shown [426] that long-term incubation with PA increased TNAP activity in osteoblast-like cell line, MOB 3-4. It has been proposed that LPA, acting via its LPA1 cell surface receptor, is able to induce cell membrane bleb [288], the process that may be related to MV formation, mineralization and apoptosis. In addition, it has been reported that LPA production in response to ATP is necessary to trigger osteogenesis [289]. LPA1 deficient mice showed craniofacial dysmorphism attributed to abnormal development of the facial bones [305]. Moreover, LPA1-deficient osteoblasts were characterized by lower differentiation potency *in vitro*[306].

### 8.4. Presence of PLD in Osteoclasts and Possible Roles

The role of PLD in osteoclasts is best exemplified under pathological conditions such as lung cancer metastasis and RA. Therefore, PLD signaling in osteoclasts is proposed as possible therapeutic strategies to prevent bone destruction. Bone is a frequent target of lung cancer metastasis that has a significant impact on morbidity [615,616]. Elevated levels of IL-8 and/or its receptors have been evidenced in cancer cells, endothelial cells, infiltrating neurotrophils and tumor-associated macrophages [617,618]. After activation of heterotrimeric small G proteins, IL-8 signaling promotes activation of the PI-3-kinase, PLC and PLD [619,620]. Exposure of human peripheral blood mononuclear cells (PBMC) to conditioned medium derived from lung cancer lines A549 and NCI-H460 as well as to sera from invasive lung cancer patients increased osteoclastogenesis in PBMC that was associated with augmented PLD activity. Depletion of IL-8 in CM derived from lung cancer lines A549 and NCI-H460 reversed the induction of osteoclastogenesis in PBMC [621]. Taken together these findings suggest that IL-8 secreted by human lung cancer cells—by increasing PLD activation—can promote osteoclast differentiation of PBMC and that PLD is involved in bone resorption by stimulating osteoclast differentiation [621]. IL-8 or IL-8-mediated PLD signaling may constitute an attractive therapeutic target for osteolytic bone metastases in lung cancer patients [621]. Under normal conditions, RANKL is produced mainly by osteoblasts and BM stromal cells. However, under pathological conditions such as RA, RANKL is also produced by T and B lymphocytes, macrophages/monocytes and synovial fibroblasts. RA synovial tissue seems be a suitable microenvironment for osteoclastogenesis since activated synovial cells and fibroblast express RANKL *in situ*[622,623]. The proinflammatory cytokine interleulin-15 (IL-15) can induce multinucleation of osteoclast-like cells in rat BM cultures [624]. IL-15 produced by RA T cells can induce osteoclastogenesis in cocultured autologous monocytes [625]. This suggests that IL-15 can mediate inflammatory bone destruction and stimulate osteoclastogenesis. IL-15 stimulation of human RA synovial fibroblasts induces simultaneous the expression of RANKL and PLD1 but not PLD2 [567]. Synovial fibroblasts treated with IL-15 induced osteoclastogenesis and PLD1 activation through the MAP kinases and NF-κB signaling pathways [567]. PLD1 may be an efficient therapeutic strategy for preventing bone destruction in RA [567].

### 8.5. Genetic Models

A recent generation of transgenic mice that do not express PLD1 [626] or PLD2 [626,627] indicated that platelets lacking PLD1 activity displayed impaired integrin activation under high shear conditions [626]. However, the skeletal formation in transgenic mice was not evaluated. The effects of silencing PLD genes on bone formation and on mineralization process were not determined.

### 8.6. Effects of PLD Metabolite at Matrix Vesicle Level

By using a fluorescence coupled-enzyme assay a phosphorylation-dependent PLD activity in MVs has been shown [628]. It is not yet known which type of PLD (PLD1 or PLD2) is predominant in MVs or what function PL has in MV. Since MV main function is to initiate HA formation, the hydrolytic activity of PLD leading to the production of PA may contribute to the mineralization process. Indeed, human alkaline phosphatase isoenzymes are able to hydrolyze phosphatidates with various fatty acyl chains (e.g., phosphatidate and dioleoyl, distearoyl, dipalmitoyl, dimyristoyl and dilauroyl phosphatidates) [614] forming P_i_. Alternatively, PA can itself alter membrane curvature and contributes to the breaking of MV membrane.

## 9. Non-HKD Enzymes—GPI-PLD

### 9.1. Groups, Subgroups and Specificity

The glycosyl-PI specific PLD (GPI-PLD) activity has been characterized and implicated in the regulation of anchoring, thereby influencing the dispersal of anchored proteins or their maintenance on the cell surface, and in this way, possibly, cell signaling [629]. The only enzyme known to date that has specificity for cleavage of the GPI anchor is GPI-PLD, which cleaves the GPI structure to generate PA and the soluble protein. Although only one GPI-PLD cDNA has been identified in mouse [630] and ox [631], two have been described in human [632]. GPI-PLD is likely to be accessible to all cells of the body due to its abundance in serum. GPI-PLD expression has been detected in several tissue or cell types such as BM, liver and islets [633–637]. A potential role of GPI-PLD during bone formation certainly depends on the presence of suitable substrates. One GPI anchored protein that has a defined role in bone mineralization is TNAP. It is possible that TNAP found in bone tissue represents a substrate for endogenous GPI-PLD, which converts it from a membrane-bound to a soluble form [634]. Other GPI-anchored molecules that may be involved in bone formation include a subset of proteoglycans, as well as glypicans, which are part of collagen framework of the highly specialized ECM of cartilaginous tissue [638].

### 9.2. Presence of GPI-PLD in Chondrocytes and Possible Roles

Glypicans expressed by chondrocytes can act as cellular modulators of responses to bone morphogenetic factors [639] and defects in the *glypican-3* gene cause an overgrowth and dysmorphic syndrome, the Simpson–Golabi–Behmel syndrome [640]. In addition, the GPI-anchored urokinase plasminogen activator receptor has been detected on the surface of chondrocytes [641], and it has been suggested that the plasminogen system may play a role in bone development by mediating effective degradation of the bone matrix. Deficiencies in this system can lead to bone overgrowth and malformations [642]. In addition, an endogenous GPI-PLD releases basic FGF-heparan sulfate proteoglycan complexes from human BM stromal cells. This mechanism of GP1 anchor cleavage could be relevant for mobilizing biologically active basic FGF in BM [633]. Gregory *et al.*[634] describe the first evidence of GPI-PLD expression during mouse embryonic ossification. GPI-PLD expression was detected predominantly at sites of skeletal development, increasing during the course of gestation. GPI-PLD was observed during both intramembraneous and endochondral ossification and localized predominantly to the ECM of chondrocytes and to primary trabeculae of the skeleton. In addition, the mouse chondrocyte cell line ATDC5 expressed GPI-PLD after experimental induction of differentiation.

### 9.3. Presence of GPI-PLD in Osteoblasts and Possible Roles

Decreasing GPI-anchored proteins by overexpressing GPI-PLD in MC3T3-E1 osteoblastic cells inhibits fluid flow induced Ca^2+^_i_ mobilization and ERK1/2 phosphorylation, suggesting that GPI-anchored proteins in cell membranes may serve as transducer to transmit fluid shear stress to biochemical responses [643].

## 10. Non-HKD Enzymes—Autotaxin

### 10.1. Groups, Subgroups and Specificity

Autotaxin (ATX, NPP2) is an ecto-nucleotide pyrophosphatase/phosphodiesterase which hydrolyzes phosphodiester bonds of various nucleotides and nucleotide derivatives [644–647]. ATX hydrolyzes various LPL including LPC [277], LPE [266] and LPS [266] leading to the formation of LPA. ATX is encoded by a single gene on human chromosome 8 whose transcription, is regulated by diverse transcription factors, results in three alternatively spliced isoforms (α, β and γ) [648]. The expression of ATX is ubiquitous. Relatively high levels of ATX are expressed in brain, kidney and lymphoid organs [648]. As a lipid mediator LPA participates in many physiological processes. It promotes platelet aggregation and thrombosis, smooth muscle contraction, anti-apoptosis wound-healing, angiogenesis, development of the nervous systems through the cell surface G protein-coupled receptor pathways [268,646,647,649]. Thus LPLs and LPA may have a significant regulatory impact on the function of cells which are primarily involved in bone formation. On the other hand, LPLs are precursors of S1P which is a potential target for RA therapies [650]. Although the major source of S1P originates from the phosphorylation of sphingosine by sphingosine kinase, a part of S1P is hydrolyzed from sphingophosphorylcholine by ATX [651] or by S1P phosphatase and S1P lyase [650]. It has been suggested that ATX may be a potential target for the treatment of patients with RA [652]. In a CIA model, treatment with type-1 SphK siRNA suppressed articular inflammation and joint destruction and down regulated S1P, IL-6, TNF-α, and IFN-γ levels [653]. S1P level in synovial fluid from RA patients is higher than that from OA patients. S1P level in serum is about 2.5 times lower than that in RA synovial fluid [654]. S1P in serum is around 600–1000 nM [655]. S1P functions frequently in inflammatory processes, but is also implicated in autoimmune diseases as well as in cellular survival, proliferation and transformation, prevention of apoptosis and stimulation of angiogenesis [650]. S1P exerts its action via two distinct pathways: 1) intracellularly as a second messager; 2) extracellularly via activating specific GPCR [650]. So far, intracellular targets of S1P have not been found although they are implicated in the regulation of cellular proliferation, suppression of apoptosis and calcium homeostasis [650]. Five GPCR have been found on the cell surface: S1P(_1–5_) [656]. The S1P_1_ was markedly expressed in synovial lining cells, vascular endothelial cells and inflammatory mononuclear cells from RA synovial tissues when compared to those from OA synovial tissues, as determined by immunostaining [654]. S1P/S1P_1_ signaling enhanced synovial cell proliferation and COX-2 induced PGE_2_ production [654] and may enhance osteoclastogenesis via RANKL expression in RA synoviocytes and CD4^+^ cells [657]. Since the inflammation in RA is related to COX-2 induced PGE_2_ production by synoviocytes and since S1P/S1P_1_ signaling may induce synovial hyperplasia and inflammation in RA, S1P/S1P_1_ signaling could be a therapeutic target in RA [657].

### 10.2. Presence of ATX in Chondrocytes and Possible Roles

The presence of ATX in chondrocytes has been ascertained for the first time during the differentiation of cells. In C3H10T1/2—a multipotential cell line with the ability to differentiate into the major mesenchymal cell types such as myoblasts, adipocytes, osteoblasts, or chondrocytes the *atx* gene was expressed during BMP2-mediated osteo-/chondrogenic differentiation *in vitro*[658]. *Atx* expression in chondrocytes has been ascertained during murine embryogenesis [659]. The involvement of an α5β1 integrin in either the cartilage differentiation program or the joint formation program has been checked by blocking α5β1 integrin. Blocking α5β1 integrin resulted in the joint formation as indicated by the induction of an ectopic joint that expressed ATX as well as *Wnt14*, the earliest joint inducer and other specific markers of joints such as *Gdf5*, chordin and CD44 [660].

### 10.3. Presence of ATX in Osteoblasts and Possible Roles

So far the presence of ATX in osteoblasts has not been documented by immunoblot analysis. However, *atx* expression in preosteoblasts and osteoblasts has been ascertained during murine embryogenesis [659]. Possible roles of ATX have been proposed from the findings based on the involvement of LPA during bone metastases. Bone metastases are frequent complications in patients suffering from different types of cancers such as breast, kidney, lung, prostate and tyroid cancers [661]. Bone metastases have two distinct features, excessive bone loss involving osteoclasts and excess bone formation involving osteoblasts. Both types of bone lesions can occur in patients with metastatic prostate cancers [662]. Most of the information on the role of ATX during bone metastases comes from the synthesis of LPA and expression of its receptors LPA_1_, LPA_2_, LPA_3_ and LPA_4_[662]. LPA can be produced as a result of tumor-cell-induced platelet aggregation, by ATX-dependent or independent expression or following by P2X7 activation in osteoblasts. [662]. Then LPA can act directly in bone cells [299,303,663]. LPA can stimulate osteoblast proliferation and differentiation as well as can stimulate platelet aggregation which may initiate an amplification loop. LPA can stimulate osteocyte dentrite outgrow that thus could contribute to inhibition of bone formation [662]. Although it has been reported that *atx* expression is elevated in cancers compared to normal tissues [277], *atx* expression in primary tumors was not correlated with the occurrence of bone-metastases over a five-year period in a cohort of 167 breast cancer patients [664]. It was proposed that stratification of patients following the breast cancer intrinsic subtype’s classification should be carried to better evaluate the relationship between ATX and bone metastases [662]. Altough the *atx* expression levels in tumor cells have been reported [277,664], the expression changes of *atx* in osteoblasts during osteosclerosis were never determined. Silencing *atx* expression in 4T1 cells impaired their capacity to form osteolytic bone metastases in immunocompetent Balb/C mice [664] suggesting that LPA—the secreted product of 4T1-cell ATX activity—may act on osteoblasts. Taken together the findings tend to suggest that LPA production is a better marker for bone-metastases than the *atx* expression level. In this respect, LPA prevents PI3K-dependent apoptosis of osteoblasts [665], promotes cytoskeletal rearrangement and cell migration [299], induces differentiation of osteoblastic MG63 cells synergically with 1α,25-(OH)_2_D_3_[295] and the osteoblastogenesis of BM stem cells [666]. Based on *Lpa1-4*-deficient mice, it was concluded that LPA may induce osteoblast differentiation through LPA_1_ and LPA_4_ receptors [304].

### 10.4. Presence of ATX in Osteoclasts and Possible Roles

Incubation of BM cells with recombinant ATX in increased significantly M-CSF/RANK-L-induced osteoclast differentiation, suggesting that LPA generated by ATX in the presence of serum might directly control osteoclast differentiation [664]. LPA can act directly on osteoclast precursors to induce their differentiation and/or on mature osteoclasts to promote survival and bone resorption activity [662].

### 10.5. Presence of ATX in Smooth Muscle Cells and Possible Roles

*Atx* expression in smooth muscle cells has been ascertained during murine embryogenesis [659]. A time-dependent increase of around 2.5 fold of ATX in the vessels has been determined by immunoblotting after ligation injury of the carotid artery in mice. Mice deficient in LPA_1_ and LPA_2_, were protected from intimal hyperplasia in response to vascular injury. This indicates that LPA may regulate vascular development and function [667].

## 11. Concluding Remarks

Critical analysis of available reports revealed that phospholipases are implicated in the mineralization process at various levels of organization of the living matter. At the molecular level, phospholipases can provide precursors of P_i_ such as phosphatidates, PChols and PEAs, that can be further hydrolysed by other enzymes in mineralizing tissues and cells. At the membrane level, the degradation of phospholipids by phospholipases can affect the structural integrity and curvature of the plasma membrane from which MVs are released as well as membranes of mature MVs in ECM, favouring deposition of calcium phosphate complex formed inside MV into ECM. Last but not least, at cellular level, phospholipases, by producing signal molecules, may modulate cellular responses of mineralization-competent cells to the signals for mineralization. Gaining further knowledge about involvement of phospholipases in the mineralization process at distinct levels can contribute to our better understanding of the molecular mechanisms of lipid degradation during physiological and pathological mineralization, and can also help to create new targets for the cure of mineralization-related human diseases.

## Figures and Tables

**Table 1:** Total lipid composition of growth plate tissues: Chondrocytes, membranes, and matrix vesicles (MVs). The cell membrane fraction represents more dense membranous material that sediments more rapidly than MVs; it probably contains some MVs that are partially calcified. It does not specifically represent the plasma membrane. Adapted from [53].

% of Total lipid					

Chondrocytes					
Lipid	Whole cartilage	Proliferating	Hypertrophic	Cell membranes	MVs
SM	8.6 ± 0.7	5.8 ± 0.4	8.0 ± 0.8	8.1 ± 0.8	13.4 ± 1.8
PC	45.2 ± 1.9	47.6 ± 1.5	38.0 ± 1.5	53.2 ± 2.2	41.8 ± 2.5
LPC	2.0 ± 0.6	1.9 ± 0.4	1.8 ± 0.4	3.5 ± 0.8	3.4 ± 0.8
PE	17.6 ± 1.0	16.9 ± 0.7	14.7 ± 0.8	14.6 ± 1.8	14.9 ± 1.8
LPE	2.0 ± 0.4	3.3 ± 0.7	2.4 ± 0.4	4.9 ± 1.2	6.5 ± 1.2
PS	5.1 ± 0.8	3.3 ± 0.3	5.0 ± 1.0	5.4 ± 0.7	9.3 ± 1.1
LPS	0.5 ± 0.2	0.2 ± 0.1	0.3 ± 0.2	2.2 ± 0.7	2.4 ± 0.8
PI	7.2 ± 0.8	6.2 ± 0.8	6.4 ± 0.8	6.1 ± 0.8	6.6 ± 0.6
LPI	1.1 ± 0.7	1.0 ± 0.6	0.5 ± 0.4	0.3 ± 0.2	1.1 ± 0.3
PA	2.0 ± 0.5	0.8 ± 0.3	1.6 ± 0.5	1.1 ± 0.2	0.9 ± 0.3
PG	1.2 ± 0.6	0.7 ± 0.3	1.2 ± 0.6	0.9 ± 0.2	1.3 ± 0.3
di-PG	3.0 ± 0.6	2.5 ± 0.4	2.9 ± 0.6	1.7 ± 0.3	1.5 ± 1.4

**Table 2:** PLA_1_ family (adapted from [69]).

Types of PLA_1_	Groups	Origin
Extracellular PLA_1_	PS-PLA_1_	Human
	mPLA_1_α	Human
	mPLA_1_β	Human
	Hepatic lipase	Human
	Endothelial lipase	Human
	Pancreatic lipase-related protein 2	Human
Intracellular PLA_1_	iPLA_1_α	Human
	iPLA_1_β	Human
	iPLA_1_γ	Human

**Table 3:** PLA_2_ family (adapted from [91]).

Type	Group	Subgroup	Origin or commun source
sPLA_2_	I	A	Cobras and kraits
	I	B	Human/porcine pancreas
	II	A	Rattlesnake/human synovial
	II	B	Gaboon viper
	II	C	Rat/murine testis
	II	D	Human/murine pancreas/spleen
	II	E	Human/murine brain/heart/uterus
	II	F	Human/murine testis/embryo
	III		Lizard/bee
	V		Human/murine heart/lung/macrophage
	IX		Snail venom
	X		Human spleen/thymus/leucocyte
	XI	A	Green rice shoots (PLA_2_-I)
	XI	B	Green rice shoots (PLA_2_-II)
	XII	A	Human/murine
	XII	B	Human/murine
	XIII		Parvovirus
	XIV		Symbiotic fungus/bacteria
cPLA_2_	IV	A(α)	Human macrophage-like U937 cells/Platelets/Raw 264.7/rat kidney, ubiquitous
	IV	B(β)	Human pancreas/liver/heart/brain/ubiquitous
	IV	C(γ)	Human heart/skeletal muscle
	IV	D(δ)	Murine placenta
	IV	E(ɛ)	Murine heart/skeletal muscle/testis/thyroid
	IV	F(η)	Murine thyroid/stomach
iPLA_2_	VI	A(β)	Human/murine
	VI	B(γ)	Human/murine
	VI	C(δ)	Human/murine
	VI	D(ɛ)	Human
	VI	E(ζ)	Human
	VI	F(η)	Human
PAF-AH	VII	A(lipoprotein-associated-PLA_2_)	Human, murine, porcine, bovine
	VII	B(PAF-AH II)	Human, bovine
	VIII	A(α1)	Human
	VIII	B(α2)	Human
Lysosomal PLA_2_	XV		Human, murine, bovine
Adipose PLA	XVI		Human,mouse

**Table 4:** Diseases and affected PLA_2_ expressions in human patients and in knockout mice.

Types of PLA_2_	Expression levels	Diseases	References
sPLA_2_-IIA	Highly expressed in synovial fluid	RA	[123,126,165,166,169]
sPLA_2_-IIA	Highly expressed in chondrocytes	RA	[126]
sPLA_2_-IID	Overexpressed in synovial fluid	RA	[124]
sPLA_2_-IIE	Overexpressed in synovial fluid	RA	[124]
sPLA_2_-V	Overexpressed in synovial fluid	RA	[124]
sPLA_2_-X	More or less expressed in synovial fluid	Active and inactive RA	[124]
sPLA_2_-IIA	Overexpressed in synovial fluid	OA	[126,167,168]
sPLA_2_-IIA	Overexpressed in VSMC	Infarctus heart	[164]
sPLA_2_-V	Overexpressed in VSMC	Infarctus Heart	[164]
cPLA_2_-α	*cPLA_2_-α*^−/−^ mice loss in function	Prevention in collagen-induced arthritis	[174]
iPLA_2_β	*iPLA_2_-β*^−/−^ mice loss in function	Low bone mass	[121]

**Table 5:** Beneficial or pathological effects of AA pathways on bone formation.

Enzymes or products or animal models	Expression level or concentration	Physiological effects	Pathological effects	References
COX-2	Increase in synovial fluid		RA	[38]
mPGES-1	Increase in synovial fluid		RA	[38]
Mice deficient in COX2	Null-COX	CIA reduction		[215]
Mice deficient in mPGES-1	Null-PGES-1	CIA reduction		[217]
PGE_2_	High level in synovial fluid		RA	[239,240]
Prostaglandin		Stimulate bone formation		[233]
Prostaglandin			Activate bone resorption in osteoporosis, RA, OA or in periodontis	[238]
PGD_2_		Stimulate osteoblast calcification		[249]
PGF_2_α		Promote osteogenic differentiation		[251]
15-Deoxy-Δ^12,14^- prostaglandin J2	Prostaglandin D2 metabolite	Activates PPARγ and TNAP expression		[249]
n-3 PUFA or conjugated linoleic acid	Exogenous addition	Beneficial effects due to modulation of COX-2		[59]

**Table 6:** Effect of cyclooxygenase (COX) inhibitors on bone formation.

Cox inhibitors	Physiological effects	Pathological effects	References
NSAIDS Ibuprofen Indomethacin		Inhibit fracture healing	[221–223]
Indomethacin		Decrease TNAP activity	[222]
NSAIDS		Decrease heterotopic calcification	[224–226]
Keterolac		Decrease in spinal fusion	[228]
COX-2 inhibitor	Decrease pain in RA		[231,241]

**Table 7:** PLC family (according to [319,343,356,357,359,368]).

Type	Group	Origin
Non-specific PLC		Mammalian
PI-specific	PLC-β PLCB1, PLCB2, PLCB3, PLCB4	Mammalian
PI-specific	PLC-γ PLCG1, PLCG2	Mammalian
PI-specific	PLC-δ PLCD1, PLCD3, PLCD4	Mammalian
PI-specific	PLC-ɛ PLCE1	Mammalian
PI-specific	PLC-η PLCH1, PLCH2	Mammalian
PI-specific	PLC-ζ PLCZ1	Mammalian
Phospholipase C-like	PLCL1, PLCL2	Mammalian
Zinc-dependent prokaryotic PLC		Bacterial
PI-DAG-lyase		Trypanosome
SMase	Neutral SMase1	Mammalian
	Neutral SMase2 (SMPD3)	Mammalian
	Neutral SMase3	Mammalian
	Lysosmal acid SMase	Mammalian
	Secreted zinc-dependent acid SMase	Mammalian
	Alkaline SMase	Mammalian

**Table 8:** PLD family (according to [77]).

Type	Variants	Origin
***PLDs with HKD motif***		
PLD1	PLD1a, PLD1b, PLD1c, PLD1d	Mammalian
PLD2	PLD2a, PLD2b, PLD2c	Mammalian
PLD3		Mammalian
Endonuclease-like mitochondrial PLD		Mammalian
***Non-HKD PLDs***		
GPI-PLD		Mammalian
N-acyl PE-PLD		Mammalian
cytochrome P450 1A2		Mammalian
cytochtome P450 2E1		Mammalian
ATX		Mammalian
